# The dual roles of RPE65 S-palmitoylation in membrane association and visual cycle function

**DOI:** 10.1038/s41598-019-41501-w

**Published:** 2019-03-26

**Authors:** Sheetal Uppal, Tingting Liu, Eugenia Poliakov, Susan Gentleman, T. Michael Redmond

**Affiliations:** 1 Laboratory of Retinal Cell and Molecular Biology, National Eye Institute, National Institutes of Health, Bethesda, MD 20892 United States; 2 Present Address: Department of Transfusion, The Affiliated Drum Tower Hospital of Nanjing University Medical School, Nanjing, 210008 P.R. China

## Abstract

Association with the endoplasmic reticulum (ER) membrane is a critical requirement for the catalytic function of RPE65. Several studies have investigated the nature of the RPE65-membrane interaction; however, complete understanding of its mode of membrane binding is still lacking. Previous biochemical studies suggest the membrane interaction can be partly attributed to S-palmitoylation, but the existence of RPE65 palmitoylation remains a matter of debate. Here, we re-examined RPE65 palmitoylation, and its functional consequence in the visual cycle. We clearly demonstrate that RPE65 is post-translationally modified by a palmitoyl moiety, but this is not universal (about 25% of RPE65). By extensive mutational studies we mapped the S-palmitoylation sites to residues C112 and C146. Inhibition of palmitoylation using 2-bromopalmitate and 2-fluoropalmitate completely abolish its membrane association. Furthermore, palmitoylation-deficient C112 mutants are significantly impeded in membrane association. Finally, we show that RPE65 palmitoylation level is highly regulated by lecithin:retinol acyltransferase (LRAT) enzyme. In the presence of all-*trans* retinol, LRAT substrate, there is a significant decrease in the level of palmitoylation of RPE65. In conclusion, our findings suggest that RPE65 is indeed a dynamically-regulated palmitoylated protein and that palmitoylation is necessary for regulating its membrane binding, and to perform its normal visual cycle function.

## Introduction

RPE65 is a critical player in the visual (retinoid) cycle that continuously regenerates 11-*cis* retinal, the chromophore of rhodopsin, and is the retinol isomerase that converts all-*trans* retinyl esters to 11-*cis* retinol^1–3^. RPE65 is highly preferentially expressed in retinal pigment epithelium (RPE). As a non-heme iron metalloenzyme with 7-bladed β-propeller architecture, RPE65 belongs to a family of carotenoid cleavage oxygenases (CCOs) but is functionally distinct from other members^3–5^. Studies with *Rpe65* knock-out mice have well established its functional significance in the visual cycle^6^. Many dozens of mutations in the *RPE65* gene have been identified in humans and are associated with hereditary childhood blinding diseases, including Leber congenital amaurosis 2 and juvenile onset retinitis pigmentosa^7,8^. Together, these findings strongly indicate that RPE65 is indispensable for normal vision.

Even before the initial functional characterisation of RPE65^9,10^, it was shown that the microsomal membrane fraction of bovine RPE exhibits retinoid isomerisation activity^11^. Later studies by Nikolaeva *et al*. showed that the association of RPE65 with the phospholipid membrane is essential for its structural maintenance and catalytic activity^12^. This work also concluded that membrane association is necessary for RPE65 to extract its lipophilic substrate, all-*trans* retinyl esters (atRE) mainly all-*trans* retinyl palmitate, from the RPE membrane^12^. Structural inspection of RPE65 reveals the presence of a long hydrophobic tunnel from the exterior to the protein’s catalytic core that may serve as a direct route for substrate entry and/or product exit^13^. Based on previous findings, different mechanisms have been proposed for RPE65-membrane interaction, including interaction via an amphipathic helix (which contains cysteine C112)^9,14,15^. One of the proposed mechanisms involves S-palmitoylation of RPE65 that would confer affinity for the membrane^16^. Indeed, early mass spectrometric analysis of intact native and expressed RPE65 suggested the presence of significant post-translational modification of the membrane-associated form of RPE65^17^; this would be consistent with palmitoylation. Protein palmitoylation, a reversible and dynamic process, involves the attachment of a saturated palmitic (C_16_) fatty acid to a cysteine residue. This post-translational modification (PTM) has been implicated in protein localisation, regulation of protein stability and activity, and promotion of stable membrane binding^18^. Three cysteine residues (C231, C329 and C330) were first reported to be the target sites for palmitoylation^16^. Later work, however, revealed that these three cysteines showed no modification by mass spectrometry (MS) analysis. Furthermore, two (C231 and C330) of the three are not conserved. Later, from structural considerations, and by biochemical observations, C112 was identified as the actual palmitoylation site^13,19^. This mode of membrane binding has, however, been challenged by another study that suggested that no post-translational palmitoylation is involved in anchoring RPE65 to the membrane, but rather that membrane association only occurs via electrostatic interactions^14^.

In the past, the identification and detection of protein palmitoylation has been technically challenging owing to the low sensitivity of the traditional radioactive methods, and the tendency for palmitoyl loss during MS sample preparation. This may have contributed to the variable findings of RPE65 palmitoylation^16^. Therefore, in the present study, we revisited the question of RPE65 palmitoylation using simple and robust detection methods that enable rapid identification of palmitoylated proteins. We performed extensive site-directed mutational analysis to map the S-palmitoylation sites on RPE65 and investigated the potential consequences of palmitoylation on RPE65’s membrane association. To validate the palmitoylation sites obtained by these biochemical methods, we also confirmed our results using a highly sensitive and quantitative mass spectrometry approach. Overall, our findings demonstrate that RPE65 is a dynamically regulated palmitoylated protein involving two potential sites (C112 and C146), rather than just one (C112) as previously suggested. We also show that palmitoylation at C112 plays an important role in membrane association of RPE65. In addition, we find that LRAT influences the dynamics of RPE65 palmitoylation, and that this may be important for normal visual cycle function.

## Results

### RPE65 is a palmitoylated protein

**Figure 1:**
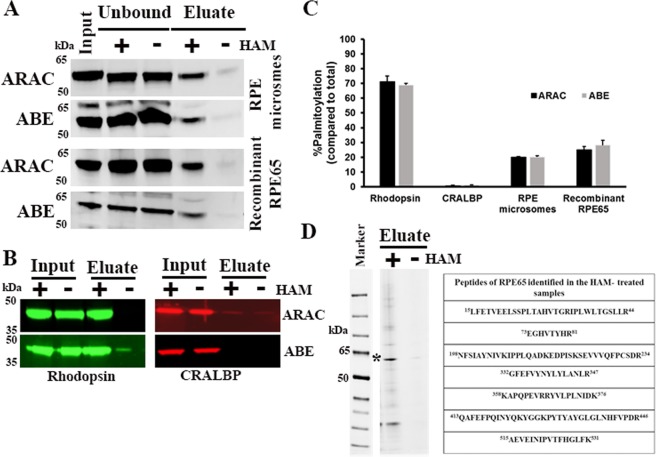
Detection of RPE65 palmitoylation. (**A**,**B**) Analysis of palmitoylation of RPE65 from RPE microsomal extracts and HEK293F-expressed lysates was performed by acyl-RAC and ABE assays. Rhodopsin and CRALBP were used as positive and negative control proteins, respectively. Samples were treated with a final concentration of 0.5 M hydroxylamine (HAM) or 0.5 M NaCl (control) and then palmitoylated proteins were enriched using beads (thiopropyl-sepharose for acyl-RAC and streptavidin-agarose for ABE) were eluted using 2.5% β-mercaptoethanol in SDS-PAGE sample buffer. Equal amounts (~20 µg) of total (indicated as “input”) and eluted protein from control (indicated as “−”) and HAM-treated (indicated as “+”) samples were separated by SDS-PAGE followed by immunoblotting with primary antibody to RPE65. Results are representative of three independent experiments. (**C**) The bar graph indicates normalized palmitoylation percentage averaged from three different experiments of Acyl-RAC and ABE assays. Error bars represents standard deviation. (**D**) Following Acyl-RAC, eluted proteins from control and HAM-treated samples separated by SDS-PAGE were stained using blazin blue protein gel stain. Asterisk shows enriched RPE65 band at ~65 kDa position in the HAM-treated sample. Box shows the identified peptides of RPE65 in the HAM-treated sample using in-gel trypsin digestion and mass spectrometry.

To test whether RPE65 undergoes palmitoylation, we used RPE65-enriched bovine RPE microsomes and HEK293F-expressing wild type RPE65 (recombinant RPE65) in two different palmitoylation assays: the Acyl-resin-assisted capture (ARAC) and Acyl-biotinyl exchange (ABE) assays. Both assays use a thiol blocker to block free cysteines followed by hydroxylamine (HAM) treatment to remove thioester-linked palmitates attached to cysteine residues and the protein is then isolated by affinity purification depending on the method. Native bovine RPE65 microsomes and recombinant expressed wild type RPE65 showed a prominent protein band in the HAM-treated sample (Fig. 1A), while it was much reduced in the untreated control sample, indicating S-palmitoylation of RPE65. For this study, a well-known palmitoylated protein, rhodopsin, and a non-palmitoylated protein, cellular retinaldehyde-binding protein (CRALBP (RLBP1)), served as positive and negative controls, respectively. As expected, our results showed a palmitoylation signal for rhodopsin (Fig. 1B). In contrast, and as expected, CRALBP was not detected as a palmitoylated protein (Fig. 1B). Quantitative analysis of palmitoylation revealed that only a small fraction of RPE65 (~25%) was modified by S-palmitoylation compared to rhodopsin with ~70% palmitoylation (Fig. 1C). As shown in Fig. 1A, acyl-RAC and ABE results showed the presence of RPE65 in the unbound fraction of HAM-treated sample. We re-incubated the unbound fraction from both control and the HAM-treated samples with fresh beads and Fig. S1 shows that the unbound fraction consists of non-palmitoylated RPE65 which does not bind to the beads. These results suggest that RPE65 palmitoylation is a dynamic or transitory process, i.e., with the concurrent existence of both palmitoylated and non-palmitoylated states. We also tested different concentrations of hydroxylamine for palmitoylation in the range of 50–500 mM and subsequent experiments were performed with 500 mM hydroxylamine concentration to achieve complete removal of palmitate from the protein (data not shown).

We further confirmed our results with the identification of RPE65 in the eluates from the acyl-RAC experiment by mass spectrometry. Eluted samples (HAM-treated and untreated) were subjected to SDS-PAGE followed by in-gel trypsin digestion (Fig. 1D). Peptides were acidified and analysed by mass spectrometry. RPE65 peptides were identified in the HAM-treated eluate, but not in the HAM-untreated control eluate (Fig. 1D). Together, these data demonstrated that RPE65 is a palmitoylated protein with the palmitoyl moiety attached to cysteines via a thioester bond.

### Identification of palmitoylated cysteine residues in RPE65

**Figure 2:**
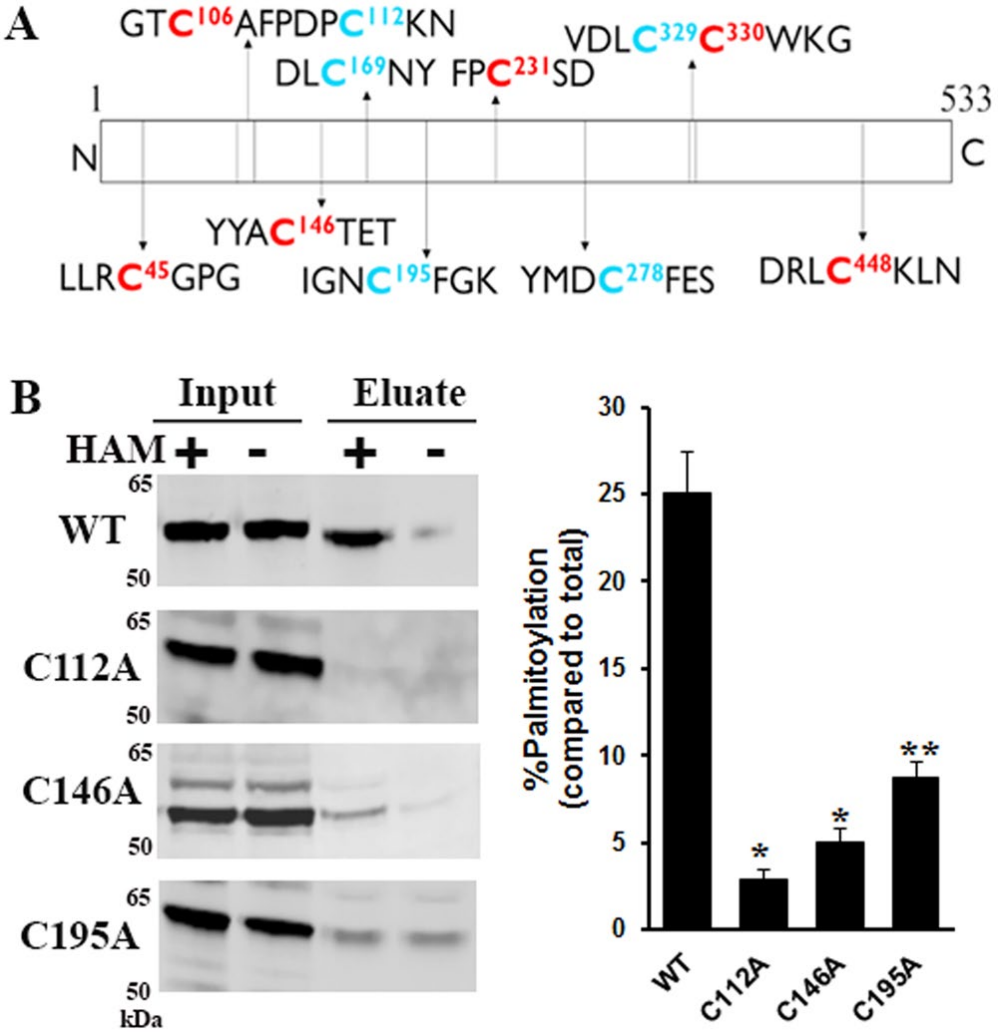
Mutational analysis of cysteine residues to identify potential palmitoylation sites in RPE65. (**A**) Schematic of dog RPE65 amino acid sequence showing cysteine residues (numbers in superscript represent the residue position). Cysteines in red and blue colour indicate the non-conserved and conserved cysteines, respectively, among different species. (**B**) Identification of palmitoylated cysteine residues. HEK293F cells overexpressing mutant C112A, C146A and C195A proteins were analysed for palmitoylation using acyl-RAC assay followed by immunoblotting for RPE65 using ~20 µg of input and pulldown eluted samples. Samples were treated with 0.5 M hydroxylamine (HAM; indicated as “+”) or 0.5 M NaCl (indicated as “−”), respectively. Results were calculated as mean ± S.D. from three independent experiments. *P < 0.0001, **P < 0.0003, unpaired student’s t-test.

No consensus sequence other than an available cysteine residue is required for a protein to undergo palmitoylation. RPE65 is 533 amino acids in length and most mammalian orthologs contain at least 11 cysteines residues, as shown in Fig. 2A. Human and bovine RPE65s both contain a 12^th^ Cys, C396, not found in other species, while human has a 13^th^ Cys, C427, also not in other species. Out of the 11 cysteines, 5 cysteines residues are perfectly conserved throughout different species (Fig. S2), a sixth, C146, is almost perfectly conserved (except in one of three paralogs in teleost fishes and in hagfish). Structural inspection of cysteines in RPE65 show that 5 cysteines (C112, C169, C195, C278 and C448) are surface-exposed with their sulfhydryl groups facing outward, whereas all other cysteines residues are buried in the protein core to a greater or lesser extent (Fig. S3). While many studies have been performed over the last decade, there is still no consensus on RPE65 palmitoylation. To determine which cysteines are palmitoylated, we performed extensive mutational studies where all conserved and non-conserved cysteine residues were substituted to alanine or serine and their effect on palmitoylation levels assessed in comparison to wild type RPE65.

All mutants were transfected into HEK293F cells and their palmitoylation levels were determined as shown in Fig. S4. Western blot analysis of acyl-RAC experiments for cysteine mutants (Ala or Ser substituted) showed that all cysteines except C112, C146 and C195 showed results similar to wild type RPE65 with signal detected only in the HAM-treated sample (Figs 2B and S4A,B). From our results, we observed that the C112 mutants show significant reduction (5–6 fold decrease) in the palmitoylation level with a very faint protein band detected in the HAM-treated sample (Fig. 2B), supporting previous findings that this residue is an actual palmitoylation site. Our results also revealed that another cysteine residue, C146, is involved in palmitoylation (Fig. 2B). We also performed ABE assays which further corroborated our results for both mutants, showing significant decrease in the palmitoylation signal compared to wild type RPE65 (Fig. S4A). However, the C195 mutant (Fig. 2B) displayed unexpected results in the acyl-RAC assay with the signal observed in both control and HAM-treated samples, indicating the presence of HAM-independent binding to the thiopropyl resin with this mutant. This result was inconsistent with the results from the ABE method that displayed only HAM-dependent binding of the C195 mutant, possibly due to the conformational changes in the structure of RPE65 induced by the mutation of C195 residue (Fig. S4B).Serine mutants for these three residues (C112, C146 and C195) in the acyl-RAC assay showed a similar palmitoylation profile to the alanine mutants (Fig. S4C). Collectively, we conclude that mutation of these three cysteine residues significantly affect the palmitoylation of RPE65 and these results also indicate that the cysteine residues 112 and 146 are palmitoylated, and thus contribute to the overall palmitoylation of RPE65 protein.

### Validation of S-palmitoylation sites in RPE65 via MS-coupled acyl exchange labeling

Mass spectrometry (MS) can provide a direct identification of palmitoylated cysteine residues in a protein. However, direct detection of labile protein palmitoylation using mass spectrometry is a challenging task because sample preparation and MS analysis often leads to palmitoyl loss, resulting in false negative results. Therefore, we combined the acyl-exchange labeling method with high definition mass spectrometry (HDMS^E^) to irreversibly replace the palmitoyl group with 4-vinyl pyridine (pyridyl ethyl; 4-VP) after treatment with hydroxylamine, distinguishing these from N-ethylmaleimide (NEM)-labelled, originally free thiol groups. The pyridyl ethyl modification is more stable than the thioester-linked palmitoyl modification.

**Figure 3:**
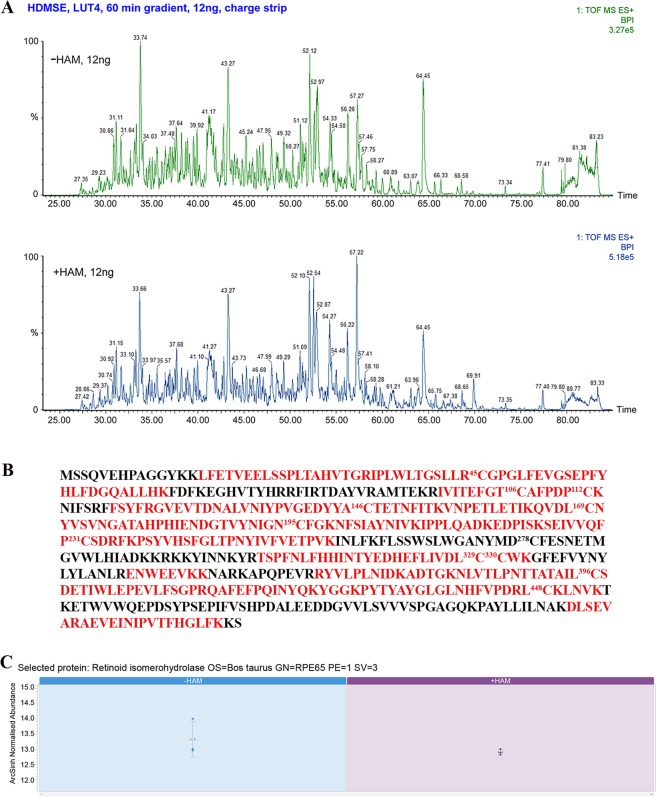
MS-coupled acyl-labeling of bovine microsome RPE65. (**A**) Screenshots of **LC-HDMS**^**E**^ runs for hydroxylamine-untreated (control) and treated samples from bovine RPE microsomes. (**B**) MS identified peptides of bovine RPE65 (in red) corresponds to >80% sequence coverage. Numbers in superscript indicate the residue positions of the different cysteines. (**C**) **LC-HDMS**^**E**^ comparison of relative abundance of total RPE65 peptides in the control and HAM-treated bovine microsomes shows that the levels of peptides were similar. The error bars show ± standard error as calculated from the three technical replicate samples.

**Figure 4:**
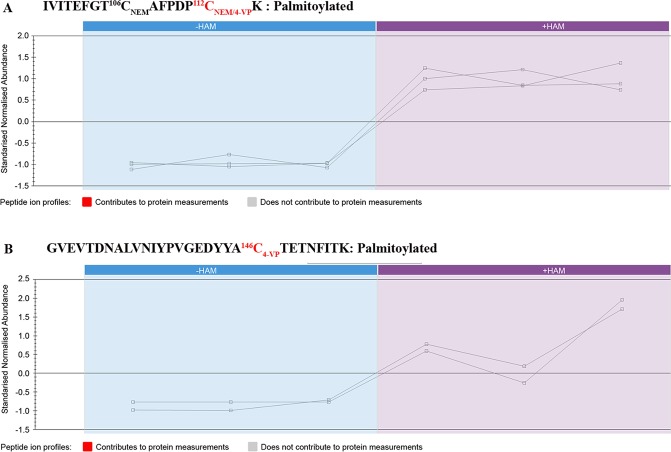
LC-HDMS^E^ comparison of relative abundance of C112- and C146-containing peptides in control and hydroxylamine-treated bovine RPE microsomes. Comparison of relative abundance of RPE65 peptides containing C112 (**A**) and C146 (**B**) in the control and HAM-treated bovine RPE microsomes. N-ethyl maleimide (NEM) and 4-vinyl pyridine (4-VP) modification represents the non-palmitoylation and palmitoylation of cysteine residues, respectively. The square boxes represent the technical repeats of the sample and the lines represent the comparative behaviour of the peptide ion of interest in the −HAM and +HAM samples.

To evaluate LC-HDMS^E^ workflow performance for identification of palmitoylated cysteines, we again used reference samples– tryptic digests of rhodopsin and CRALBP. This procedure definitively confirmed the palmitoylation of rhodopsin at two cysteines (322 and 323), while, as expected, no pyridyl ethyl modified peptides were observed in CRALBP, indicating no palmitoylation (Fig. S5). We employed the same MS methodology for the identification of RPE65 palmitoylation using bovine RPE microsomes. We treated this source of bovine RPE65 with trypsin, and then subjected the digest to UPLC followed by HDMS^E^. The chromatographic elution profiles for control and HAM-treated RPE65 samples were quite similar (Fig. 3A). However, using ion-mobility separation with an optimized HDMS^E^ approach increases the overall peak capacity allowing label-free quantification via a data independent acquisition (DIA) approach. Accordingly, we identified a total of 100 peptides with >80% RPE65 protein sequence coverage (Fig. 3B). Both HAM-treated and untreated (control) samples showed the same level of identified peptides (Fig. 3C). We found that almost all cysteine residues were NEM-modified, except for C112 and C146 which were pyridyl ethyl-modified. Interestingly, we observed both NEM- as well as pyridyl ethyl- modification of C112 containing peptide. Quantitative analysis revealed that these modified peptides are highly enriched in HAM-treated samples in comparison to control samples (Fig. 4A; Table 1). The high abundance (6.77; Table 1) of the NEM/NEM peptide containing C112 may be related to the specific conformation/properties of this peptide given its location overlapping the highly mobile loop of RPE65. In contrast, in Supplemental Table S1 the majority of peptides show a Max fold change of between 1 and 2 (this can be either −HAM/+HAM or +HAM/−HAM, depending on the peptide). It is possible that its observed yield was enhanced by the NEM modification in combination with HAM treatment. However, it is not clear how the difference in treatment of 0.5 M HAM (experimental) and 0.5 M NaCl (control) would have had an effect on downstream processing of the protein. This may reflect more the efficiency of detection than such a large difference of the C112 NEM/NEM peptide between the two groups and so might be due to a variation in elution efficiency and/or ionization efficiency of peptides between two groups that would ultimately affect the detection and, thus, estimation of the peptides. Additionally, the high hydrophobicity of the palmitoyl moiety can affect how palmitoyl-modified peptides, and potentially other peptides, are eluted in the HAM-untreated samples. Another possibility is the possible presence of residual NEM during hydroxylamine cleavage. This could result in the NEM alkylation of the newly exposed C112 peptide, prior to 4-vinyl pyridine treatment, thus contributing to its overall abundance in the HAM-treated samples. In the case of C146, we observed only the pyridylethyl-modified peptide to be abundant only in the HAM-treated samples (Fig. 4B; Table 1). However, we also analyzed the same samples using a different MS instrument as shown in Fig. S6, which detected only pyridylethyl-modified C112 and NEM-modified C146 peptides. The discrepancy might be due to the insufficient fragmentation or non-sufficient peptide elution from chromatographic system which might have prevented the identification of some specific peptides. Interestingly, the peptide containing C195 residue was found to be NEM-modified, confirming the ABE assay finding that C195 residue is not palmitoylated. Together, we combined the data from multiple experiments and all the identified peptides containing NEM- or pyridyl ethyl-modified cysteines are shown in Table 2. Dual modification of the C112 and C146 residues thus confirms the notion that palmitoylation of RPE65 is a dynamic process. From the mutational analysis and mass spectrometric results, we conclude that the two cysteines at 112 and 146 are target sites for palmitoylation in RPE65.

**Table 1:** LC-HDMS^E^ data parameters of peptide ions containing cysteines 112 and 146.

Peptide	Modifications	m/z	Charge	Retention time (min)	Max fold change (+HAM/−HAM)	Highest mean condition	Lowest mean condition	Normalized Abundance (+HAM)	Normalized Abundance (−HAM)	Anova (p)
IVITEFGTC^106^AFPDPC^112^K	NEM; 4-VP	657.65	3	58.036	8.18	+HAM	−HAM	1808.2	220.9	0.03722
IVITEFGTC^106^AFPDPC^112^K	NEM; NEM	995.97	2	64.397	6.77	+HAM	−HAM	17756	2619.9	0.00260
GVEVTDNALVNIYPVGEDYYAC^146^TETNFITK	4-VP	887.93	4	57.43	13.3	+HAM	−HAM	3426.9	256.8	0.00832
GVEVTDNALVNIYPVGEDYYAC^146^TETNFITK	NEM	1154.2	3	64.088	1.45	+HAM	−HAM	11785.5	8116.9	0.374*

Cysteine residues are underlined, with superscript number showing residue position. 4-VP modification on cysteine indicates palmitoylation. This data was excerpted from Supplementary Dataset 1.

*Not significant.

**Table 2:** Tryptic peptides of bovine RPE65 identified using MS-coupled acyl exchange labeling method.

Identified peptides of RPE65	Cys Modification
C^45^GPGLFEVGSEPFYHLFDGQALLHK	NEM
IVITEFGTC^106^AFPDP**C**^**112**^K	**106: NEM** **112: NEM; 4-VP** ^**#**^
GVEVTDNALVNIYPVGEDYYA**C**^**146**^TETNFITK	**4-VP (NEM** ^**#**^ **)**
QVDLC^169^NYVSVNGATAHPHIENDGTVYNIGNC^195^FGK	169: NEM 195: NEM^#^
SEIVVQFPC^231^SDR	NEM
KFLSSWSLWGANYMDC^278^FESNETMGVWLHIADK	n.d
TSPFNLFHHINTYEDHEFLIVDLC^329^C^330^WK	329, 330: NEM
NLVTLPNTTATAILC^396^SDETIWLEPEVLFSGPR	NEM
PYTYAYGLGLNHFVPDRLC^448^K	NEM

Cysteine residues are underlined, with superscript number showing residue position. Cysteine residues in **bold** were modified by 4-VP indicating palmitoylation as detected using Synapt G2-Si HDMS^E^. Modification marked with # in superscript was identified using AB Sciex6600 w/SelexION mass spectrometer. NEM: N-ethyl maleimide; 4-VP: 4-vinyl pyridine.

### LRAT mediates the regulation of RPE65 palmitoylation

**Figure 5:**
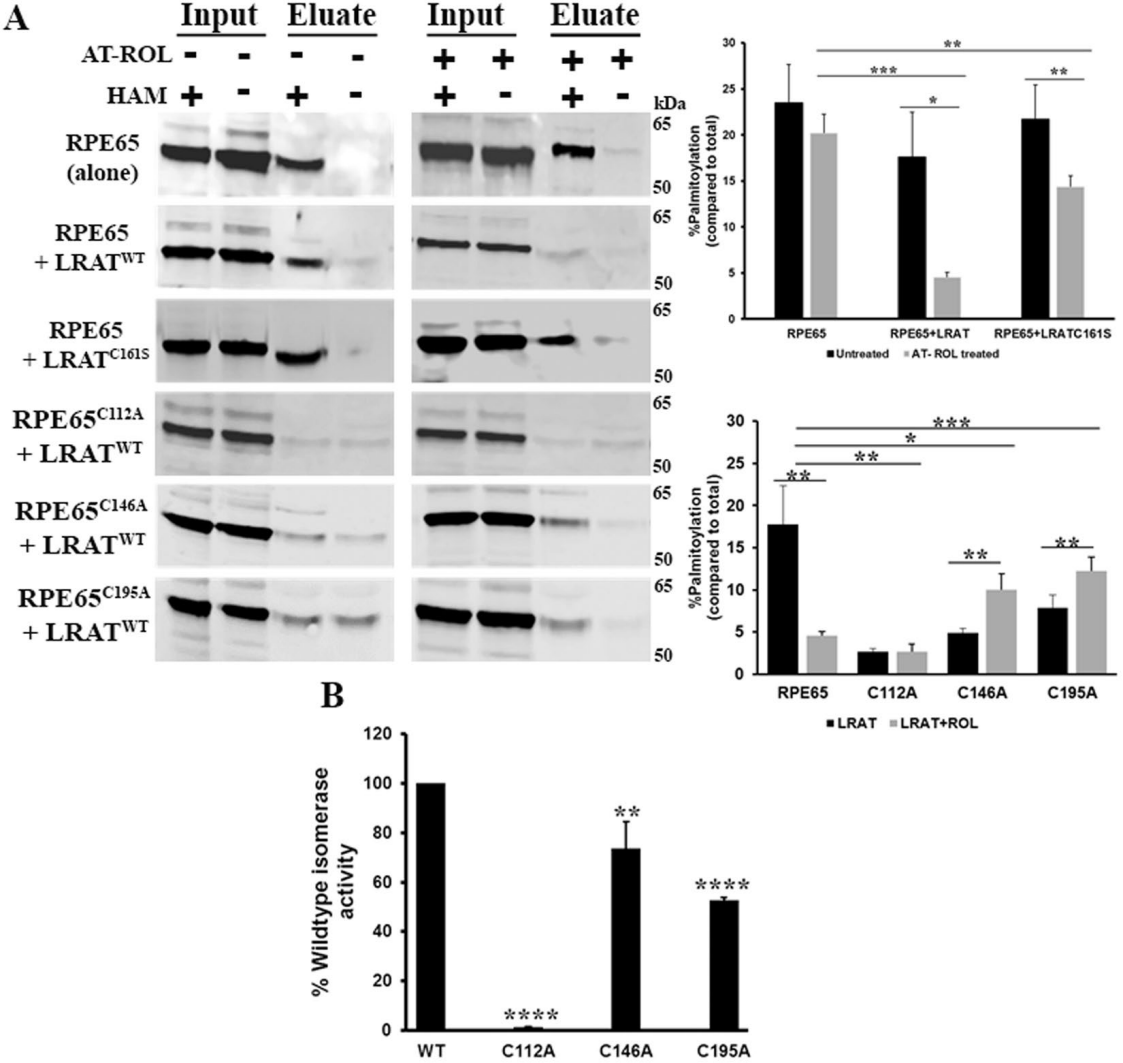
Palmitoylation levels and isomerisation activity of RPE65 wild type and cysteine mutants in *in vitro* minimal visual cycle system. (**A**) HEK293F cell cultures transfected with pVitro2/RPE65 wild type or cysteine mutants + CRALBP and pVitro3/LRAT or LRAT^C161S^ mutant + RDH5 vectors were incubated with 2.5 μM all-*trans* retinol (at-ROL) for 7 h. Cell extracts were analysed by acyl-RAC assay to monitor changes in the palmitoylation status of RPE65. Samples were treated with 0.5 M hydroxylamine (HAM; indicated as “+”) or 0.5 M NaCl (indicated as “−”), respectively. (**B**) *In vitro* isomerase activity was assessed for wildtype RPE65 and cysteine mutants (C112, C146 and C195). Data were represented as mean ±S.D. from three independent experiments. *P < 0.01, **P < 0.05, ***P < 0.0005 and ****P < 0.0001 unpaired and paired student’s t-test.

Redmond *et al*.^3^ demonstrated that the presence of LRAT markedly enhances the isomerisation activity of RPE65 and proposed that LRAT might facilitate the release of bound palmitoyl (palmitate) product from RPE65 (and also suggested by Xue *et al*.^16^) to restore it to its active state. To provide a possible explanation for this, we investigated whether LRAT has an effect on RPE65 palmitoylation using a heterologous non-RPE cell culture system that mimics visual cycle as established by Redmond *et al*.^3^. We co-transfected HEK293F cells with pVitro2/RPE65+ CRALBP and pVitro3/LRAT+ retinol dehydrogenase 5 (RDH5) and their expression was analysed by western blotting (Fig. S7). When co-expressed with wild type LRAT, western blot analysis did not show any significant change in the palmitoylation signal as compared to RPE65 alone in the HAM-treated sample (Fig. 5A). Similar results were observed when the LRAT^C161S^ mutant^20^, which exhibits 30–40% of WT catalytic activity, was used (Fig. 5A). Next, we performed the same experiments in the presence of 2.5 µM all-*trans* retinol (at-ROL; LRAT substrate), a concentration close to the calculated physiological concentration of 3.8 µM in the retina following a 2% fractional bleach of rhodopsin, and used for our minimal visual cycle culture system^3^. We observed a significant decrease in the level of palmitoylation of RPE65. However, this decrease was reversed when the hypomorphic LRAT^C161S^ mutant was used (Fig. 5A). These results clearly demonstrate that RPE65 palmitoylation is modulated by LRAT enzymatic activity. This may allow efficient palmitate recycling from RPE65 and thus increase the overall efficiency of the visual cycle.

Moreover, we analysed the palmitoylation level of cysteine mutants in the active visual cycle system by co-expressing cysteine mutants of RPE65 with LRAT and treated with at-ROL. Acyl-RAC results showed slight increase in the palmitoylation signal of C146 and C195 mutants, however, C112 showed very faint or almost no palmitoylation signal in the HAM-treated sample (Fig. 5A). This result led us to conclude that C112 is the critical residue involved in the palmitoylation of RPE65. Furthermore, we think that mutation of C146 and C195 residues slow down the palmitate recycling efficiency and thus affect the enzymatic activity of RPE65. To test this hypothesis, we investigated the influence of these cysteine mutations on the isomerisation activity of RPE65. Isomerase assays show that both C146 and C195 mutants are still active, however, the level of 11-*cis* retinol production from all-*trans* retinol is ~20–50% lower than the wild type RPE65 (Fig. 5B) which indicates that these residues might contribute in the efficient release of bound palmitate derived from the substrate, at-ROL. In contrast, the mutants of C112 do not show any isomerisation activity (Fig. 5B) and thus are catalytically inactive.

### Palmitoylation at C112 is required for membrane localisation of RPE65

**Figure 6:**
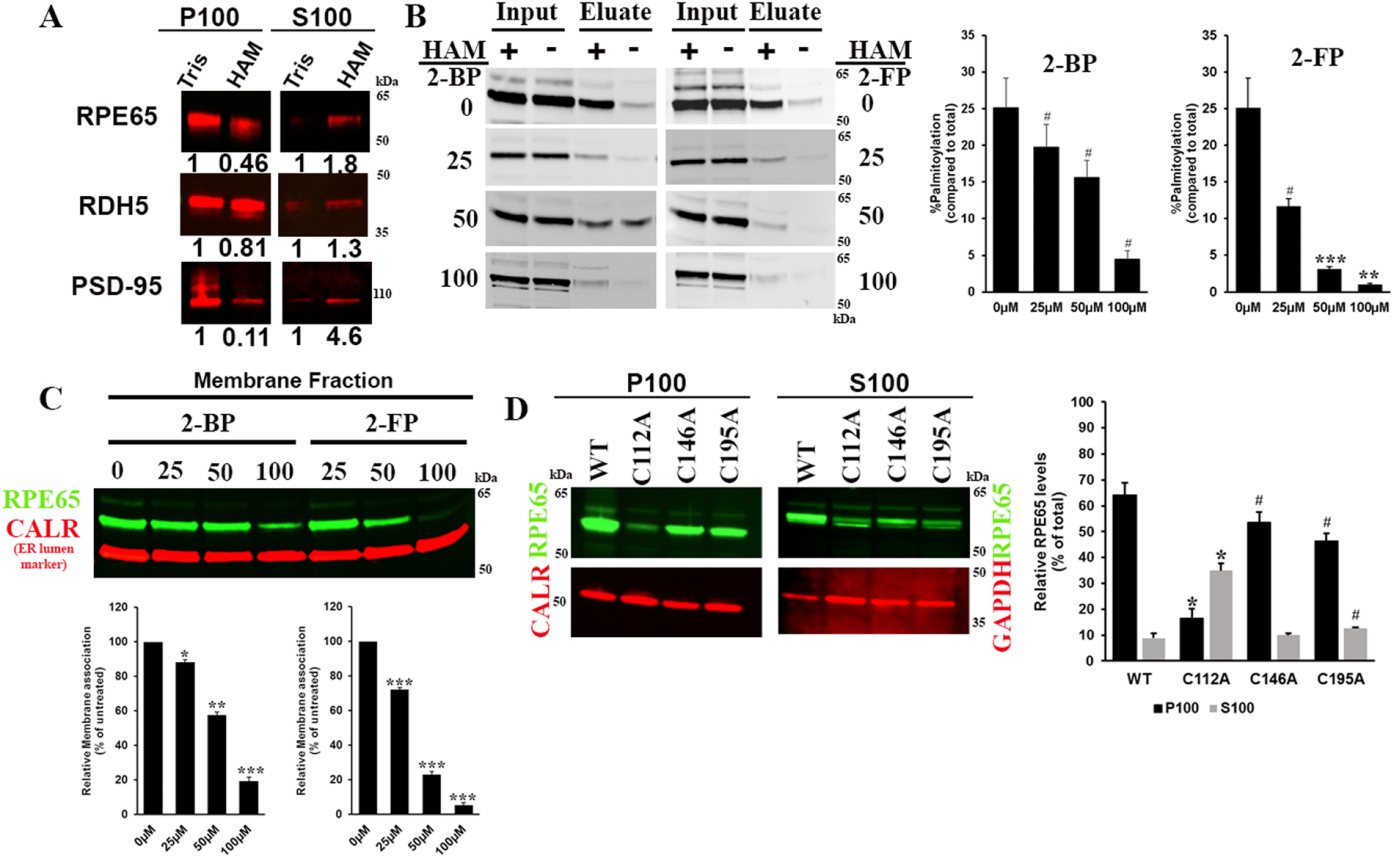
Palmitoylation at C112 is crucial for RPE65-membrane interaction. (**A**) Bovine RPE and rod outer segments (ROS) membrane fractions were treated with 0.1 M Tris (control) or 0.5 M HAM to remove RPE65 palmitoylation, followed by membrane sedimentation. The distribution of RDH5, a non-palmitoylated transmembrane protein and PSD-95, a palmitoylated peripheral membrane protein (positive control) are indicated. P100 (membrane) and S100 (soluble) fractions were analyzed by western blotting using anti-RPE65, anti-PSD-95, and anti-RDH5 antibody. The numbers indicate the levels normalized to control sample. HEK293F-cells overexpressing RPE65 were treated with the global protein palmitoylation inhibitors 2-bromopalmitate (top) and 2-fluoropalmitate (bottom). Suppression of RPE65 palmitoylation was analyzed by acyl-RAC assay, which showed dose-dependent decrease of RPE65 palmitoylation (**B**) and sucrose subcellular fractionation was performed to assess the membrane localisation (**C**) of RPE65 after treatment with palmitoylation inhibitor (**D**) Wildtype RPE65 and cysteine mutants (C112, C146 and C195) were analysed for membrane association using sucrose subcellular fractionation. Protein amount used for immunoblotting was ~20 µg. GAPDH and calreticulin were loading controls for cytosolic and membrane fractions, respectively. Samples were treated with 0.5 M hydroxylamine (HAM; indicated as “+”) or 0.5 M NaCl (indicated as “−”), respectively. Data were represented as mean ±S.D. from three independent experiments. *P < 0.001, **P < 0.001, ***P < 0.0005 and ^#^P < 0.05 unpaired and paired student’s t-test.

As palmitoylation is essential for membrane association of several proteins, we determined the effect of reducing or eliminating palmitoylation on the membrane association of RPE65. We examined RPE65 membrane association in native bovine RPE microsomes after removal of the palmitoyl group by HAM. Our results show that there is a considerable decrease in the RPE65 in the membrane fraction upon 0.5 M HAM treatment (Fig. 6A). In contrast, the transmembrane RDH5 protein did not show any change in the distribution after HAM treatment (Fig. 6A). As a positive control, we monitored the redistribution following 0.5 M HAM treatment of postsynaptic density protein 95 (PSD-95) protein, a peripheral synaptic membrane protein that also localizes to the mammalian retina^21^, and which solely depends upon palmitoylation for its association with the membrane^22^. We observed a significant release of PSD-95 protein from the pellet fraction (Fig. 6A) and concomitant increase in the PSD-95 levels in the supernatant fraction. However, we did not observe complete loss of RPE65 from membrane after HAM treatment which suggests that palmitoylation is not entirely responsible for RPE65-membrane affinity. We also treated HEK293F cells expressing RPE65 with different concentrations of 2-bromopalmitate (2-BP) and 2-fluoropalmitate (2-FP)^23,24^, potent inhibitors of protein palmitoylation, and performed acyl-RAC assays to determine the changes in palmitoylation. Our results showed that palmitoylation of RPE65 gradually decreased with increasing dose of inhibitors, compared with DMSO-treated control samples (Fig. 6B). Upon treatment with 100 µM of either inhibitor a 5–6 fold decrease in palmitoylation was found in RPE65. We observed a significant reduction in the membrane-bound RPE65 level (Fig. 6C) at 100 µM concentration suggesting an essential role of palmitoylation in facilitating the interaction of RPE65 with membranes.

Next, we tested the membrane localisation of palmitoylation-defective C112, C146 and C195 mutants in active visual cycle system. No change in the expression level of cysteine mutants were observed in comparison to wild type RPE65 as shown in Fig. S8 indicating that mutation of these three cysteines residues did not affect the RPE65 protein expression. As expected, wild type RPE65 is present predominantly in the microsomal membrane fraction (Fig. 6D). Western blot analysis showed that C146 and C195 mutants still retained their membrane localisation while the C112 mutant showed almost complete lack of membrane association (Fig. 6D). In the case of C112 mutants, we did not see any increase in the redistribution from membrane to cytosolic fraction. Our results corroborate the previous findings of Takahashi *et al*. who showed that C112A mutant proteins were present in the fraction containing cytoskeletal or inclusion body proteins and that this might have been due to the misfolding of C112A mutant proteins^19^. A possible explanation for this is that mislocalised and improperly folded C112 mutant proteins in the cytosolic fraction are less stable and thus more prone to rapid degradation compared to wild type RPE65. It is well established that lysosome-mediated degradation of misfolded proteins is a major pathway of proteostasis, in addition to proteasome-mediated degradation^25^. For this, we checked the lysosomal fraction for wild type RPE65 and C112 mutant proteins and found that C112 mutant protein accumulated in lysosomes, whereas very low wild type RPE65 protein is present (Fig. S9). The absence of RPE65 on membrane correlates with the loss in the activity of C112 mutants (Fig. 5C) which further strengthens the idea that the capacity for membrane association is a necessary requirement for RPE65 to be a functionally active retinoid isomerase. Overall, these data suggest that palmitoylation at C112 is essential for membrane association.

## Discussion

Early studies^9,10^ clearly demonstrated that RPE65 is a membrane-associated protein though it lacks any transmembrane segments. While several studies suggest that palmitoylation plays a role in RPE65 membrane association^19,26^, the existence of RPE65 palmitoylation has also been challenged^14^. In addition, palmitoylation of RPE65 has been invoked as a molecular switch regulating its function^16^, but many of the premises of this proposal have been widely questioned and/or disproven. To address this ongoing debate in RPE65 biochemistry, in the present study we have determined the palmitoylation status of RPE65 using widely accepted robust methods, the acyl-RAC and ABE assays, and corroborated this by mass spectrometry. Further, we have investigated the mechanism of regulation of RPE65 palmitoylation and elucidate its functional significance in the visual cycle.

The present study confirms that RPE65 is post-translationally modified by cysteine palmitoylation via thioester bond. However, in contrast to recent previous studies^19,26^, we find multiple sites of RPE65 palmitoylation. Following extensive site-directed mutagenesis of conserved and non-conserved cysteines, we show that palmitoylation occurs at cysteines 112 and 146; substitution at either site significantly lowers the palmitoylation level of RPE65. We further confirmed these palmitoylation sites using an improved LC-MS protocol that provides better protein coverage with high quantitative accuracy. However, we observed a discrepancy in the quantitative analysis of the palmitoylation level of RPE65 from biochemical pulldown assays and mass spectrometry results. It is possible that the pulldown methods are relatively insensitive to palmitoylation at the C146 residue, which is located in the substrate cleft. Moreover, the heterogeneous labeling of C112 in palmityolated RPE65 is consistent with a role in membrane targeting and may reflect the activity of acyl protein thioesterases on this surface-exposed residue. In contrast, C146 located in the substrate cleft appears to be consistently palmitoylated. However, the status of C195 is unclear, although the acyl-RAC assay results suggest that it has a unique role in RPE65 compared with any of the other cysteines. From structural analysis, it is evident that the C195 residue is located in a highly mobile loop (C195-A203) at the opening of the substrate cleft. This loop is not well resolved in any of the inhibitory ligand-bound structures of RPE65, which are the only crystals in which palmitate is seen coordinated with the Fe center^27^. The thiol group of C195 exists in two different conformations, in one conformation it is solvent exposed and in the other the thiol group is facing toward the catalytic core (Fig. S10). We suggest that, in the uninhibited state of palmitoylated RPE65, the palmitate is moved from the Fe center to C146, and that C195 may play an indirect role in this process. Thus, we conclude that RPE65 is a doubly palmitoylated protein which is in disagreement with prior findings that suggest that C112 is the sole palmitoylation site. Even earlier, in contrast, Xue *et al*.^16^ concluded that RPE65 was triply palmitoylated at C231, C329 and C330; however, none of these residues we (present study) or others^19,26^ have determined to be palmitoylated.

Our data also reveals the dynamic nature of RPE65 palmitoylation-depalmitoylation, highlighting the existence of alternate potential structural conformations of RPE65. This suggests that the functional role of RPE65 palmitoylation is rather different from merely maintaining the physical location of domains of the protein, such as in the case of rhodopsin. The primary function of palmitoylation in many peripheral membrane proteins such as synaptosomal-associated protein 25 (SNAP25), cysteine-string protein (CSP) and postsynaptic density protein 95 (PSD-95) with no obvious membrane targeting motifs, is to modulate interactions with membranes^22,28,29^. Similarly, our results and those of others clearly show that palmitoylation at C112 is crucial for membrane anchoring of RPE65. In contrast to this, Jin *et al*.^30^ found in *Lrat* knockout mice, where RPE65 palmitoylation might not be predicted to occur, that membrane association of RPE65 is still found. Also, they determined that 100 µM 2-bromopalmitate did not prevent RPE65 attachment to membranes, contrary to our findings. Additionally, we also show that 100 µM 2-fluoropalmitate abolishes membrane association. Though not as widely used as 2-BP, 2-FP has been shown to inhibit palmitoylation of myelin proteolipid protein (PLP1)^23^, as well as inhibiting fatty acyl:CoA ligases (ACSLs) and sphingosine metabolism^31^. Furthermore, while Jin *et al*.^30^ did not see any effect of 100 µM 2-BP on RPE65 isomerase activity, we previously showed that 2-fluoropalmitate was an inhibitor of RPE65 (IC_50_ = ~70 µM)^32^. These findings are not necessarily contradictory, in light of the clearly complex interactions of RPE65 with the RPE ER membranes.

As reported earlier, the C112 residue is located in the highly mobile loop consisting of residues 109–125 that is not fully resolved in any of the crystal structures. Predicted by secondary structure algorithms^10,27^ to be an amphipathic helix, this region may act as an intrinsic membrane binding region in RPE65 such that the predicted amphipathic helix mediates a membrane affinity which may be further tightened by C112 palmitoylation. In addition, there are other regions (aa196–201, aa234–236, and aa261–271) that, with aa109–125, contribute to a hydrophobic patch on RPE65 (Fig. S11) that may play an important role in RPE65 membrane affinity. Furthermore, amphipathic helices have been shown to direct palmitoylation in the electrogenic sodium/calcium exchanger1 (NCX1)^33^ and in the host-mediated S-palmitoylated Legionella E3 ubiquitin ligase effector protein GobX^34^. In the case of NCX1, it is suggested that the hydrophilic face of the amphipathic helix is recognized by the cellular palmitoylation machinery^33^. We are currently investigating the intriguing possibility that a similar situation occurs in RPE65. We found, similar to previous findings^20^, that the C112 mutants showed no membrane attachment and also no catalytic activity. which give rise to another possibility that palmitoylation may not only be important for stable anchoring on membrane, but is also required for initial targeting of RPE65 to membrane. In contrast, both C146 and C195 mutants retain their membrane localisation as well as partial retention of their isomerisation activities, unlike C112 mutants. These results suggest that C146 may be involved in functions other than targeting RPE65 to membrane, such as a catalytic function. C146 might facilitate the release of bound palmitate from the RPE65 active site which requires a change in the conformation of RPE65 that is different from the conformation seen in the crystal structure (PDB ID: 4RSC). In the crystal structure, the palmitate is coordinated to the Fe centre with the C146-thiol being 8 Å away from the coordinated palmitate (Fig. S12). We propose that the origin and role of palmitoylation at C112 and C146 are quite different in nature (Table 3), a point which needs further investigation in detail. In this regard, C112 could be palmitoylated enzymatically via DHHCs (Aspartate-Histidine-Histidine-Cysteine domain-containing palmitoyltransferases). On the other hand, we also cannot rule out the possibility of palmitoylation of C112 because of its close proximity to the membrane phospholipids and their acyl chains. In the case of the C195 residue, the C195A mutant shows an anomalous result in comparison to other cysteine mutants and has reduced catalytic activity and reduced overall palmitoylation. However, the mass spectrometry results show only NEM modification of C195, indicating that C195 itself is not palmitoylated. Thus, we conclude that C195 may have an effect on the palmitoylation of C112 and/or C146.

**Table 3:** Difference between C112 and C146 residues in RPE65 protein.

	Cysteine 112 (C112)	Cysteine 146 (C146)
Residue location in the 3-D structure	located in surface-exposed highly mobile loop	located in hydrophobic substrate-binding cleft
MS-coupled acyl exchange labeling identified modification	±Palmitoylation (shows NEM and 4-VP modification)	+Palmitoylation (only 4-VP modification)
Membrane binding of RPE65	significantly affect membrane binding	No role in membrane binding
Catalytic activity of RPE65	mutation abolishes activity	mutation reduces activity
Proposed source of palmitoyl moiety	DHHC^38^ -mediated palmitoylation using palmitoyl-CoA	Substrate mediated palmitoylation

In 2004, Rando and coworkers^16^ proposed that the function of RPE65 is regulated by a “palmitoylation switch mechanism” whereby a triply palmitoylated membrane-associated form of RPE65 (“mRPE65”) served as a chaperone for all-*trans* retinyl esters, making this substrate available for the then putative and unidentified retinol isomerohydrolase (IMH), and that “mRPE65” also was a palmitoyl donor to LRAT in the dark. When all its palmitoyls had been transferred, RPE65 then reverted to a soluble form (“sRPE65”) that served as a chaperone for all-*trans* retinol. The switch mechanism (“sRPE65” to “mRPE65”) operated when LRAT activity (in the light) increased all-*trans* retinyl ester levels and LRAT then possibly served as a palmitoyl donor to restore “sRPE65” to “mRPE65”. However, absent from this proposed mechanism was the possibility that RPE65 actually was the IMH, an already credible premise based on the biochemical phenotype of the *Rpe65* knockout mouse^6^. (The premise that RPE65 was the IMH was fully verified biochemically shortly thereafter^1–3^). In addition, none of the cysteines (C231, C329 and C330), identified by labeling with tritiated palmitic acid, are actually ones found to be palmitoylated in subsequent studies, including the present one. Despite these serious drawbacks of their proposed mechanism, our data corroborates the possibility of palmitate transfer from RPE65 to LRAT in a more complete experimental system using full-length LRAT, as suggested by their experiments showing apparent transfer of palmitate from RPE65 to truncated LRAT^16^. Based on these findings, we speculate that the palmitoyl groups may constitute a “conserved” transferable entity in the visual cycle. It can be conjectured that conservation of palmitoyl groups reduces the energy requirement and/or enhances the efficiency of the visual cycle. Aside from corroborating, along with others, the genuine occurrence of palmitoylation on RPE65, our data suggest that RPE65 is, in actual fact, capable of transferring palmitoyl moieties to LRAT. Using our HEK293F-based *in vitro* visual cycle system expressing key visual cycle proteins^3^ to study the integration of RPE65 palmitoylation into the overall visual cycle, we show that LRAT plays a crucial role in the regulation of palmitoylation turnover on RPE65. We speculate that the bound palmitoyl is removed by LRAT to be used for esterification of all-*trans* retinol, and concomitantly readying RPE65 for the next round of reaction in rapidly generating 11-*cis* retinol. This would support one of the conclusions of the Xue paper^16^, that palmitoyl groups are capable of being transferred between RPE65 and LRAT.

In conclusion, our findings provide a better understanding of the functional importance of RPE65 palmitoylation in the visual cycle. Our findings specify that: (1) RPE65 is a dynamically-regulated palmitoylated protein in that palmitoylated and unpalmitoylated populations of RPE65 co-exist, (2) C112 palmitoylation is necessary for regulating RPE65’s membrane binding to perform its normal visual cycle function, and (3) the palmitoylation level of RPE65 is highly modulated by LRAT. This latter point suggests that LRAT may play a role, not only in providing RPE65’s obligate substrate but also in regulating the extent of palmitoylation of RPE65 by affecting the level of available palmitoyl groups. The significance of the latter possibilities are currently under study.

## Materials and Methods

### Preparation of bovine RPE microsomes

Bovine eyes were obtained from a local slaughterhouse (J.W. Treuth and Sons, Catonsville, MD). After removal of the anterior section, vitreous and retina, RPE cells were collected by gentle brushing into extraction buffer (0.32 M sucrose in 0.1 M phosphate buffer, pH 7.4 containing 1× complete protease inhibitor cocktail). RPE cells were homogenized by 10–12 strokes in a Dounce glass homogenizer and centrifuged at 30,000 × g for 30 min at 4 °C to remove cell debris (unbroken cells, nuclei, mitochondria, lysosomes, and melanin granules). The resulting supernatant was centrifuged at 100,000 × g for 1 h at 4 °C to sediment the microsomal membrane fraction. The membrane pellet was resuspended in 0.1 M phosphate buffer, pH 7.4, containing complete protease inhibitor cocktail and stored at −80 °C until further use. For biochemical assays, bovine RPE microsomal fraction samples were resuspended in phosphate buffered saline (PBS; 137 mM NaCl, 4.3 mM Na_2_HPO_4_, 1.47 mM KH_2_PO_4_) containing 5 mM EDTA, 0.3% CHAPS and complete protease inhibitor cocktail.

### Site-directed mutagenesis of RPE65

Site-directed mutagenesis was carried out by using the QuikChange XL or QuikChange Lightning site-directed mutagenesis kits (Agilent, CA) to generate RPE65 with the indicated Cys- to -Ala or -Ser substitutions. Mutagenic primers were designed as per the manufacturer’s protocols and primer sequences are given in Table S1. The plasmid pVitro2 (Invivogen, San Diego, CA) containing the dog RPE65 gene ORF was used as the template for mutagenesis. The mutant and wild type plasmids were purified by Qiagen plasmid purification kits (Qiagen, Germantown, MD) and mutations were confirmed by DNA sequencing.

### Cell culture and transfection

Cell culture methods and transient transfection protocols were used as previously published (3). Briefly, HEK-293F (HEK293F) FreeStyle (Invitrogen) were grown in serum-free FreeStyle 293 expression medium (Invitrogen) with shaking at 130 rpm in a 37 °C incubator under 8% CO_2_. The HEK293F cells were transfected using 2 ml of OptiMem-I reduced serum medium containing 40 µl of HEK293Fectin reagent (Invitrogen) with 20 µg of each expression plasmid. Cells were harvested after 48 h transfection. In some experiments, all-*trans* retinol (Sigma, St. Louis, MO) was added 24 h post-transfection to a final concentration of 2.5 µM, the cells were cultured for a further 7 h, and then harvested by centrifugation for retinoid analysis.

### In-gel trypsin digestion and MALDI-TOF mass spectrometry analysis

The selected protein spot was excised (with a clean new scalpel blade) from a wet gel stained with blazin’ blue (GoldBio, St. Louis, MO), and the gel pieces placed into a clean 1.5 ml plastic microcentrifuge tube. The gel pieces were destained with a freshly prepared wash solution (50% methanol and 5% acetic acid), overnight at room temperature. The destaining solution was removed, and the gel pieces were washed with 200 µl water (3 times), dehydrated with 200 µl of 100% acetonitrile (ACN) until the gel pieces turned an opaque white colour, and dried in a vacuum centrifuge for 5 min. The gel pieces were rehydrated with 200 µl of 100 mM ammonium bicarbonate for 10 min at room temperature, an equivalent volume of trypsin (Trypsin Gold, mass spectrometry grade (Promega, Madison, WI); 20 ng/µl) was added, and digestion was performed overnight at 37 °C. After digestion, the peptides were extracted (2x) with 50 µl of 50% ACN in 5% trifluoroacetic acid (TFA; v/v) with occasional gentle vortex mixing for 10 min. The supernatants were pooled, and were dried in a vacuum centrifuge. For matrix-assisted laser desorption ionisation (MALDI)-MS analysis, the dried tryptic digest samples were reconstituted with 10 µl of 0.1% TFA, and were purified with a ZipTip_C18_ (Millipore, Billerica, MA) using the procedure recommended by the manufacturer. The purified peptides were eluted from ZipTip using 50–80% ACN and mixed in 1:1 ratio with a solution of α-cyano-4-hydroxycinnamic acid (CHCA; 10 mg/ml; Fluka Chemie GmbH, Steinheim, Germany) and alkylated dihydroxybenzoic acid (ADHB; 5 mg/ml; Sigma) prepared with 1:1 ratio in 50% acetonitrile:0.1% TFA (vol/vol). The mixed solution was spotted onto a MALDI target plate and allowed to dry at room temperature. The tryptic peptides were analyzed by a MALDI-time of flight (MALDI-TOF) method (AB Sciex TOF/TOF 5800, AB Sciex, Ontario, Canada) in a reflector positive ion mode. Mass spectra and data-dependent tandem mass spectra (MS/MS) were collected and all spectra were internally mass-calibrated with the protonated molecule ions, (M + H)^+^, of trypsin autodigestion peptides (*m*/*z* 515.33, 842.51, and 2211.10) and matrix peaks (*m*/*z* 379.11 and 568.14). Mascot 2.5 Software (Matrix Science, Boston, MA) was used for data analysis.

### Retinoid extractions and HPLC

Retinoids were extracted and saponified under dim red light as previously described^35^ from cells harvested by centrifugation. Resultant isomeric retinols were analyzed on 5-μm particle Lichrospher (Alltech, Deerfield, IL) normal phase columns (2 × 250 mm) on a HPLC system equipped with a UV-visible diode-array detector (Agilent 1100/1200 series, Agilent Technologies, New Castle, DE), following Landers and Olson^36^ as modified by us^3,35^. Data were analyzed using ChemStation32 software (Agilent).

### Preparation of HEK293F membrane fractions

HEK293F cells transfected with plasmids were washed with ice-cold PBS, resuspended in lysis buffer (50 mM HEPES pH7.4, 150 mM NaCl, 5 mM EDTA, 1 mM PMSF, and 1× complete protease inhibitor cocktail (Roche Diagnostics, Indianapolis, IN) and homogenized by 10–12 strokes in a Dounce glass homogenizer. After removal of cell debris by centrifugation at 800 × g for 10 min at 4 °C, cell lysates were centrifuged at 20,000 × g for 30 min at 4 °C. The resulting membrane pellet was suspended in lysis buffer containing 0.1% Triton X-100 and was used for biochemical assays.

### Subcellular protein fractionation

Cells were disrupted using N_2_ cavitation under high pressure (700 pound per square inch) in a solution of 0.33 M sucrose in 10 mM phosphate buffer, pH 7.4, containing complete protease inhibitor cocktail. The lysates were centrifuged at 300 × g for 10 min at 4 °C to remove cell debris and nuclei. The post-nuclear supernatant was then centrifuged at 30,000 × g for 20 min at 4 °C to separate large organelles and plasma membrane. The supernatant was collected and centrifuged at 100,000 × g for 1 h at 4 °C. The resulting microsomal pellet was resuspended in 0.3% CHAPS in phosphate buffer (10 mM, pH 7.4) containing complete EDTA-free protease inhibitor cocktail. The supernatant was taken as the “cytosolic fraction”. Fractions were subjected to western blotting using antibodies to RPE65, GAPDH (cytosolic marker) and calreticulin (ER marker).

### Detection of S-palmitoylation of RPE65

For Acyl-RAC analysis, 500 µg of HEK293F membrane fraction or 200 µg of bovine RPE microsomal membrane fraction was used. To block free SH groups, 2X blocking buffer (0.1 M HEPES, 1 mM EDTA, 2.5% (v/v) SDS and 0.5% (v/v) S-methyl methanethiosulfonate (MMTS; Sigma)) was added to the resuspended proteins and incubated for 15 min at 40 °C with frequent vortexing. Subsequently, three volumes of ice-cold 100% acetone was added to the blocking protein mixture and incubated for 30 min at −20 °C and then centrifuged at 10,000 × g for 10 min at 4 °C to pellet precipitated proteins. The pellet was washed three times in 1 ml of ice-cold 70% (v/v) acetone in H_2_O and resuspended in buffer A (0.1 M HEPES, 5 mM EDTA, 1% SDS (v/v)). A fraction of the solubilized pellet was saved as the input. The remainder was treated with hydroxylamine (HAM; Sigma) to cleave thioesters, and the free cysteine residues captured by thiopropyl Sepharose beads (Sigma). Briefly, 2 M HAM was added to ~200 µg together with the beads (previously activated for 15 min with dH_2_O and equilibrated with buffer A) to a final concentration of 0.5 M HAM and 10% (w/v) beads. As a negative control, 2 M NaCl (final concentration = 0.5 M NaCl) in HEPES buffer was used instead of HAM. These samples were then incubated for 2 h at room temperature with end-over-end mixing. After brief centrifugation at 3000 × g, the supernatant was removed and retained as the “unbound” fraction. The remaining beads were washed five times with 1 ml buffer A. Subsequently, the proteins were eluted from the beads by incubating with 50 μl Laemmli sample buffer (LSB:BME:Buffer A 0.9:0.1:3) for 15 min at room temperature and then 5 min at 95 °C. The eluted proteins were the “bound” fraction. Fractions were separated by SDS-PAGE and analysed by western blot. Experiments were repeated at least three times.

Palmitoylation of RPE65 and its specific cysteine mutants was also assayed by the ABE method, as described previously with minor modifications^37^. Briefly, harvested cells were resuspended in buffer B (50 mM Tris, pH 7.4; 150 mM NaCl; 5 mM EDTA; 0.1% SDS; 10 mM N-ethylmaleimide (NEM; Sigma)) containing complete protease inhibitor cocktail. Cells were homogenized by 30 strokes in a Dounce glass homogenizer on ice and incubated at 4 °C for 1 h with end-over-end mixing after addition of 1.7% Triton X-100. The lysate was centrifuged at 250 × g for 5 min and the clarified supernatant recovered. Following this, protein precipitation was performed using the chloroform/methanol (CM) method. The pellet was resuspended in 4SB buffer (4% SDS, 50 mM Tris, 5 mM EDTA, pH7.4) containing 1 mM NEM and incubated at 37 °C for 10 min, with occasional agitation, diluted 4-fold with buffer B containing 1 mM NEM, 0.2% Triton X-100 and 1 mM PMSF and the sample incubated overnight with end-over-end rotation at 4 °C. NEM was then removed by three sequential steps of CM precipitation. Following the third precipitation, the protein precipitate was divided into two equal aliquots. One aliquot was mixed with freshly prepared 1 m HAM pH 7.4, 1 mm N-[6-(biotinamido)hexyl]-3′-(2′-pyridyldithio) propionamide-biotin (Thermo Scientific Pierce, Waltham, MA), 0.2% Triton X-100 (Sigma), and 1× complete protease inhibitor cocktail, and the other aliquot was treated with the identical mixture except that it did not contain hydroxylamine. Both aliquots were then incubated for 1 h at room temperature with rotation. The proteins were precipitated by the CM method and treated with 200 μm N-[6-(biotinamido) hexyl]-3′-(2′-pyridyldithio)propionamide-biotin, 0.2% Triton X-100, and 1× protease inhibitor cocktail at room temperature for 1 h. The N-[6-(Biotinamido)hexyl]-3′-(2′-pyridyldithio) propionamide-biotin was then removed by three sequential CM precipitations. Following the third precipitation, proteins were immunoprecipitated with streptavidin-agarose (Thermo Scientific Pierce) and eluted with SDS-PAGE loading buffer containing 2.5% β-mercaptoethanol by boiling for 5 min. Samples were then subjected to western blot analysis with anti-RPE65 antibody. Experiments were repeated at least three times.

### Acyl-exchange labeling and mass spectrometry

To evaluate palmitoylation in RPE65, we used RPE65-enriched bovine RPE microsomes. In our experimental approach, samples were treated with NEM to block reduced cysteines. Next, one half of the sample was treated with hydroxylamine (HAM) to cleave any Cys-palmitoyl thioester bonds, whereas the other half was the control sample. Both samples were then subjected to SAGE-electrophoretic lateral fractionation (SageELF, Sage Science, Beverly, MA) using a 5% agarose SDS gel to size-fractionate the protein sample into 12 different fractions that are recovered in SDS gel buffer. The fractions containing RPE65 were pooled, verified by western blotting, and concentration/buffer exchange was carried out using Amicon® Ultra-0.5 ml centrifugal filter devices (Millipore NMWL = 30 kDa). Following concentration, the HAM-untreated/treated protein samples were treated with 20 mM DTT or TCEP, and then with 0.5 M 4-vinyl pyridine to label the free thiol groups generated after HAM treatment, distinguishing these from original NEM-labeled thiol groups. Trypsin (10 ng; Trypsin Gold, mass spectrometry grade 1 µg/µl (Promega)) was added to the reduced alkylated samples and these were incubated overnight at 37 °C. Rhodopsin and CRALBP, fractionated and processed similarly, were used as positive and negative controls, respectively. Approximately 12 ng of trypsin digested HAM-untreated and treated samples of RPE65 and control proteins were separated using liquid chromatography using a 15 cm × 75 µm HSS T3 C_18_ reverse phase column. Solvent A was 0.1% trifluoroacetic acid in HPLC grade water. The column gradient used was 3% to 85% acetonitrile with 0.1% formic acid (solvent B) over 90 min with a flow rate of 300 nl/min. The trapping configuration was 3 min with a flow rate of 10 µl/min using 99.9% solvent A. The effluent from the LC was directed to a Synapt G2-Si HDMS^E^ mass spectrometer (Waters Corp., Milford, MA) equipped with ion-mobility separation. Triplicate HDMS^E^ injections were performed using modified loading based on scouting runs, and triplicate HD-DDA runs were performed with double or triple the load used for HDMS^E^ experiments. Peptide identification was done using Progenesis QI software for proteomics (NonLinearDynamics, Durham, NC) that enables label-free analysis for quantification and identification of proteins in complex samples via a data-independent approach.

### Global Inhibition of palmitoylation with 2-bromopalmitate (2-BP) and 2-fluoropalmitate (2-FP)

Stock solutions of 100 mM 2-BP (Sigma) and 36 mM 2-FP (Cayman Chemicals, Ann Arbor, MI) were prepared in DMSO. Briefly, 24 h after transfection, HEK293F cells expressing WT RPE65 were incubated with different concentrations of inhibitor and incubated for an additional 8 h. Control cells were treated with DMSO only. Cells were harvested and used for further biochemical experiments.

### Protein determination and immunoblot analysis

Total protein content in cell lysate was estimated by advanced protein assay reagent (Cytoskeleton Inc., Denver,CO) with bovine serum albumin (Sigma) as a standard. Samples were combined with 4 × LDS sample buffer, denatured samples were separated by SDS-PAGE using 4–12% gradient BisTris NuPAGE (Invitrogen) and electro-transferred to nitrocellulose membrane using iBlot2 gel transfer device (Thermo Fisher Scientific, Waltham, MA). Membranes were blocked with Odyssey blocking buffer (LI-COR, Lincoln, NE) for fluorescent western blotting and then probed with primary antibodies in Odyssey blocking buffer for overnight at 4 °C. The membranes were washed three times for 5 min each with 1 × TBS containing 0.1% Tween 20, incubated with IRDye 680RD and 800CW secondary antibodies (1: 15,000) in Odyssey blocking buffer for 1 h at 37 °C and then washed three times with 1 × TBS containing 0.1% Tween 20. For detection, membranes were scanned on an Odyssey Infrared Imager (LI-COR Biosciences) in the 700 nm and 800 nm channels. The primary antibodies used were as follows: rabbit anti-RPE65 antibody (1:2,000); rabbit anti-LRAT antibody (1:2,000); rabbit anti-RDH5 antibody (1: 2,000); rabbit anti-CRALBP antibody (1:20,000; gift of John Saari, University of Washington, Seattle); goat anti-calreticulin (1:1000); rabbit anti-PSD-95 (1:1000); mouse anti-GAPDH (1:10,000); and rabbit anti-cathepsin D (1:1000).

### Data analysis

For quantification of S-palmitoylation for wild type and cysteine mutant proteins, we calculated the ratio of protein eluted in +HAM sample to total amount of protein in input sample. The software Image Studio™ Lite V3.1 (LI-COR Biosciences) was used for measuring the protein density according to manufacturer’s instructions. The mean of the measured values was presented with the standard deviation of the mean (SD).

## Supplementary information

Supplemental Figures

Supplementary Dataset 1
