# Supplementary material for: The dual roles of RPE65 S-palmitoylation in membrane association and visual cycle function
Source: Sci Rep. 2019 Mar 26;9:5218. doi: 10.1038/s41598-019-41501-w (PMC6435699; doi:10.1038/s41598-019-41501-w)
Supplement: Supplementary file 1 — Supplemental Figures [file 41598_2019_41501_MOESM1_ESM.pdf]

The dual roles of RPE65 S-palmitoylation in membrane association and visual cycle function

Sheetal Uppal[1], Tingting Liu[1,2], Eugenia Poliakov[1], Susan Gentleman[1], and T. Michael Redmond[1*]

[1]Laboratory of Retinal Cell and Molecular Biology, National Eye Institute, National Institutes of Health, Bethesda, MD 20892, United States

[2]Present address: Department of Transfusion, The Affiliated Drum Tower Hospital of Nanjing University Medical School, Nanjing 210008, P.R. China

*Address correspondence to: redmondd@helix.nih.gov; Tel.: 301-496-0439; Fax: 301-402-1883.

Supplemental Figure Legends

Figure *S1*. **Co-existence of palmitoylated and non-palmitoylated populations of RPE65**. Acyl-RAC analysis of RPE65 showed that a major population of RPE65 did not pull down with thiopropyl-sepharose beads (unbound fraction). The palmitoylation status of this unbound RPE65 was checked by twice sequentially re-incubating unbound fraction (unbound fraction 1 and 2) with fresh beads, followed by immunoblot analysis using anti-RPE65 antibody. This showed that there was no pulldown of this population of RPE65, confirming its lack of palmitoylation.

Figure *S2*. **Amino acid sequence alignment of RPE65 from different species.** Sequence alignment shows the position of non-conserved (in blue color) and conserved (in red color) cysteine residues among different species. Note that only in one of three paralogs in teleost fishes and in hagfish RPE65 is C146 not completely conserved. There are 12 cysteine residues in bovine RPE65. Dog RPE65 has 11 cysteine residues, with substitution of the non-conserved cysteine 396 (present in human and bovine RPE65s) to an arginine residue.

Figure *S3*. **Structural view of surface exposed cysteine residues.** Surface view of dog RPE65 structure was visualized and generated using PyMol software. Close inspection of the cysteine residues on the three-dimensional structure of RPE65 revealed five cysteines (C112, C169, C195, C278 and C448, as shown in red color; highlighted in circle) that have their thiol groups facing to the solvent. The iron (Fe) atom in the center of the RPE65 structure is represented by orange color.

Figure *S4*. **Identification of cysteine residues involved in RPE65 palmitoylation.** HEK293F cells were transfected with cysteine mutants of RPE65 to determine the residues that undergo palmitoylation. Cell lysate was prepared (as described in Materials and Methods section) and used for ABE and acyl-RAC analysis. (A) ABE result of cysteine 112, 146 and 195 mutants compared to wild type RPE65. (B) Acyl-RAC results for both alanine and serine substituted mutants of cysteine residues other than C112, C146 and C195 showed a protein band in the HAM-treated samples and so did not affect palmitoylation of RPE65. (C) Serine substituted C112, C146 and C195 residues showed reduced or no RPE65 band in the HAM-treated samples, and thus are involved in RPE65 palmitoylation. Samples were treated with 0.5 M hydroxylamine (HAM; indicated as "+") or 0.5 M NaCl (indicated as "-"), respectively. Results were calculated as mean ± S.D. from three independent experiments. *$P<0.005$, **$P<0.05$, unpaired student's t-test.

Figure *S5*. **Mass spectrometric analyses of control and hydroxylamine-treated samples for rhodopsin and CRALBP.** Comparison of relative abundance of NEM- and 4-VP modified peptides in the control and HAM-treated samples for rhodopsin (A) and CRALBP (B). N-ethyl maleimide (NEM) and 4-vinyl pyridine (4-VP) modification represents the non-palmitoylation and palmitoylation of cysteine residues, respectively. We identified the peptide containing 4-VP modifications of cysteines 322 and 323 from the bovine rhodopsin sample. In the case of CRALBP, only NEM-modified peptides were detected, indicating lack of palmitoylation. The square box represents the technical replicates of the sample) and the line represent the comparative behavior of the peptide ion of interest in the – HAM and + HAM samples.

Figure *S6*. **MS-coupled acyl-labeling of bovine microsome RPE using different MS instruments, Synapt G2-Si HDMS, AB Sciex 6600 w/SelexION**. Rhodopsin and CRALBP were used as positive and negative control. NEM- and and 4VP-modified cysteine indicates indicates non-palmitoylation and palmitoylation of cysteine residue, respectively. The ratio of modified peptide shown was relatively high in hydroxylamine-treated samples compared to untreated samples.

Figure *S7*. **Immunoblot analysis of proteins in HEK293F-based heterologous visual cycle system.** HEK293F cells transfected with pVitro2/RPE65+CRALBP and pVitro3/LRAT or LRAT^{C161S} mutant+RDH5 plasmids were analysed by western blotting for RPE65, CRALBP, LRAT, and RDH5.

Figure *S8*. **Expression profiles of wild type and C112, C146 and C195 mutant RPE65 protein.** HEK293F cells were transfected with pVitro2/RPE65 wild type or cysteine mutants +CRALBP and ~10µg of total protein were analysed by western blotting for expression analysis. The same samples were then subjected to subcellular fractionation shown in Figure 6D.

Figure *S9*. **Presence of C112 mutant protein in lysosomal fraction.** HEK293F cells transfected with pVitro2/RPE65 wild type or C112 mutants +CRALBP were subjected to lysosomal extraction kit (Sigma) and then the lysosomal fractions were analysed by western blotting for RPE65 and cathepsin D (lysosomal marker protein).

Figure *S10*. **Enlarged view of the catalytic core of RPE65 showing rotamer variability of cysteine 195.** Structural representation of RPE65 (PDB ID: 4RSC; chain A) in complex with the non-retinoid inhibitor emixustat and palmitate. Iron (Fe) atom in the center is represented in orange color. Residues C146 and C195 are denoted as stick figures on the cartoon to mark the orientation of the thiol group. The thiol group (marked with arrows) of C195 exists in two different conformations Asterisks (*) represent the location of the unresolved loop (residues G196-S201) in the ligand bound RPE65.

Figure *S11.* **Surface view of crystal structure of RPE65.** A, RPE65 structure showing three hydrophobic regions. The region consisting of aa109-125 is generated by the ITASSER server (shown in yellow). B, hydrophobic surface view of RPE65 as predicted by PyMOL server. Red color indicates the hydrophobic surface.

Figure *S12*. **Enlarged view of the catalytic core of RPE65 showing the approximate distance of 8 Å between the bound palmitate and C146 thiol atom.** The dashed yellow line represent the measured distance between the bound palmitate and C146-thiol atom in the palmitate-bound crystal structure of RPE65 (PDB ID: 4RSC).

Figure *S13*. **Original western blot panels with gel markers for Figures 1A, 1C, 2B, 5A, 5B, 5C, 6, S1, S2, S3, S6A, S6C, S8, and S9.** Complete scanned gels for western blots shown. Red dashed line identifies cropped region shown in respective figure.

Figure *S1*

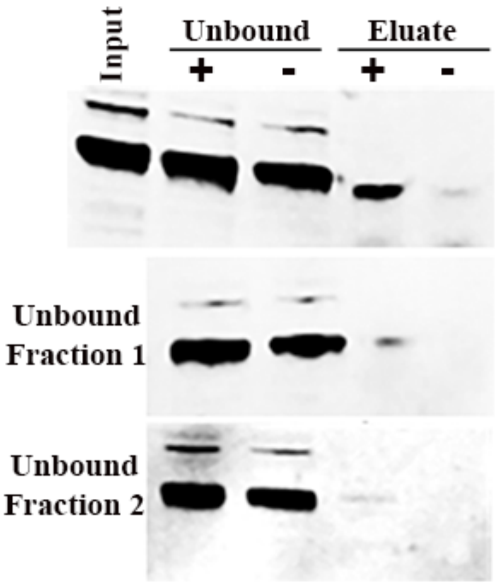
Input
Unbound
Eluate
+
-
+
-
Unbound
Fraction 1
Unbound
Fraction 2

Figure *S2*

```
                                                      45
BOVINE   1 MSSQVEHPAGGYKKLFETVEELSSPLTAHVTGRIPLWLTGSLLRCGPGLFEVGSEPFYHLFDGQALLHKFDFKEGHVTYHRRFIRTDAYVRAMTEKRIVI 100
HUMAN    1   I                                                                                                  100
DOG      1                                                                                                      100
MOUSE    1   I I                                                                                                100
CHICK    1   Y                      V         T  R              A                             V                 100
SALAM    1  TNR D           ST   VA V  Q      V  S                Q              E  G   I         T             100
ZEBRFI   1  V RF         I   A   NE    T      SFIK     L      A A            M     SN Q   F K VK       I    V   100
LAMPRY   1  ATC     K  TA Y  AQ VKT MPTQ Q    S  S   M L          E L            EISN A   AC  LK  T       N  A  100
HAGFI    1 M-YCF   S    A Y     TENSVPTTIQ H  D  N     L   C        L        M    IRK     TS  LL  S              99
               106   112                               146                    169                       195
BOVINE 101 TEFGTCAFPDPCKNIFSRFFSYFRGVEVTDNALVNIYPVGEDYYACTETNFITKVNPETLETIKQVDLCNYVSVNGATAHPHIENDGTVYNIGNCFGKNF 200
HUMAN  101                                    V                  I                                              200
DOG    101                                                       I                                              200
MOUSE  101                        K                              I                I            S                200
CHICK  101      Y Y               K           V                  I  D           K            V                  200
SALAM  101      F             L   Q L         V              Y   I       V K        I  V       H             H  200
ZEBRFI 101        Y               K       C   V  I   F  V    Y     VD    L K  M    NI  V       R          M  GA 200
LAMPRY 101      V Y               QKI I              F           I       VAK    K I I          R             GL 200
HAGFI  101      F Y                 L I   T   V  I   SF S    YV     D    LGK    D CVI  V       A   I         RM 199
                                        231                                         278
BOVINE 201 SIAYNIVKIPPLQADKEDPISKSEIVVQFPCSDRFKPSYVHSFGLTPNYIVFVETPVKINLFKFLSSWSLWGANYMDCFESNETMGVWLHIADKKRKKYI 300
HUMAN  201                                                                                                    L 300
DOG    201                         V                                    L                                       300
MOUSE  201 TV    I     K       N   V                                                          S      V     R  F 300
CHICK  201  L    IR          MN   V                                    L                            V E  KGRLL 300
SALAM  201 AF                  N AKV       E                     Q              I           H      M V E HTGE L 300
ZEBRFI 201  L     R   T K  S   E  KV     SAE           M E  F           L    A  IR S        D EK T I   R HPGE   300
LAMPRY 201  F    I     LK  VN LN MSVA  L   E           M E  F    Q      W    A GPR  T       HH     V V   R GE   300
HAGFI  201 NFV   IR   M D     LK LKV TS   RE           KSE  F    Q      W    A GPR S        D IV T I V   HSGEVL 299
                                     329330                                                              396
BOVINE 301 NNKYRTSPFNLFHHINTYEDHEFLIVDLCCWKGFEFVYNYLYLANLRENWEEVKKNARKAPQPEVRRYVLPLNIDKADTGKNLVTLPNTTATAILCSDET 400
HUMAN  301                     NG                                                                               400
DOG    301        S            N                                                S                       T R     400
MOUSE  301                                                       R  M              T   V   R      H     T R     400
CHICK  301  I     A         F           T                 A  D    Q E      A       R              Y     T R     400
SALAM  301  I     A            H                                 RS E P            D H V       N  Y     V R     400
ZEBRFI 301 DY F   AMG      C   S  IVF   A           W     A      R  MI          I  DPFREEQ   IS   Y     TMRA G  400
LAMPRY 301 DIRF AAA  I      F KEGHIVM V        I         M     L R  Y             I F EEEY     R GD     T RN G  400
HAGFI  301 PTT    S  I        EDGQIVA       Y         S   DK  NLKNCALE             HLEE EF     RVQH     T RH G  399
                                                         448
BOVINE 401 IWLEPEVLFSGPRQAFEFPQINYQKYGGKPYTYAYGLGLNHFVPDRLCKLNVKTKETWVWQEPDSYPSEPIFVSHPDALEEDDGVVLSVVVSPG-AGQKP 500
HUMAN  401                           C                                                                          500
DOG    401                                                                                                      500
MOUSE  401                          F                   K          I M               Q                          500
CHICK  401 V      I     H         K         T                                                      I I    S P   500
SALAM  401                        K H   D      V          S                T         Q   I           I    E     500
ZEBRFI 401                        RMVN  N                I     R                 L  QT  GVD    ILMTI       - R  499
LAMPRY 401                    R   E NN RD  F            KIY        H    I GT       IAR   QD     L TT     P  RT  501
HAGFI  400 V               D      KRCN  N               KIY       QML  HT  T       IAR  GTE     LVTS    A S  S  499

BOVINE 501 AYLLILNAKDLSEVARAEVEINIPVTFHGLFKKS--- 533
HUMAN  501                                       533
DOG    501                                       533
MOUSE  501     V        I      T           R     533
CHICK  501           M          V          RA    533
SALAM  501  F        M  I     DS        M   A    533
ZEBRFI 500 T C          I       LT      MY P-    531
LAMPRY 502  F         T       DV    S   M KE SKH 536
HAGFI  500  F  L D R  T L     DR        M  T-    532
```

Figure *S3*

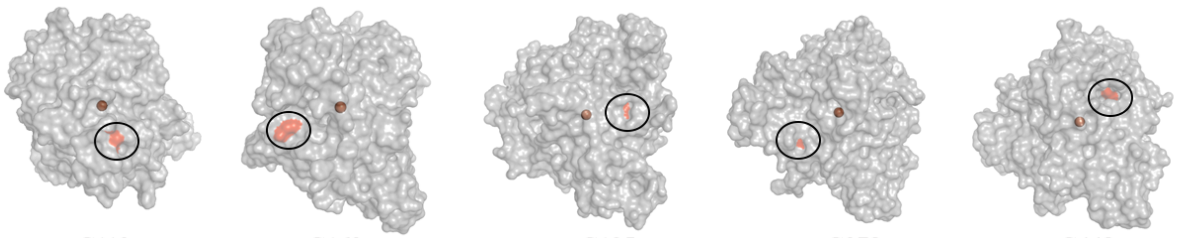

C112 **C169** **C195** **C278** **C448**

Figure *S4*

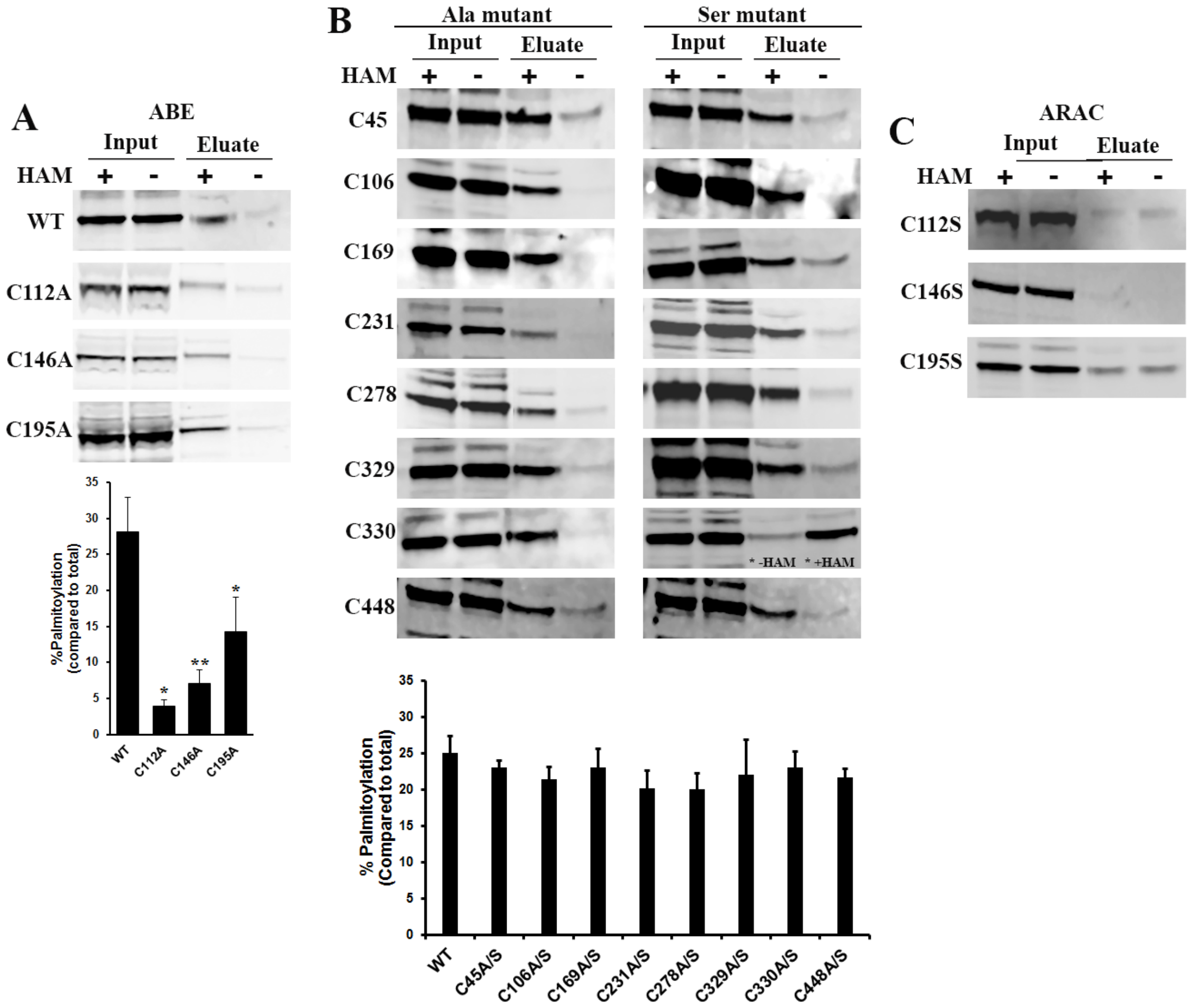
A
ABE
Input
Eluate
HAM
+
-
+
-
WT
C112A
C146A
C195A
%Palmitoylation (compared to total)
B
Ala mutant
Ser mutant
Input
Eluate
C45
C106
C169
C231
C278
C329
C330
C448
* -HAM
* +HAM
% Palmitoylation (Compared to total)
WT
C45A/S
C106A/S
C169A/S
C231A/S
C278A/S
C329A/S
C330A/S
C448A/S
C
ARAC
C112S
C146S
C195S

Figure *S5*

A Rhodopsin: QFRN^{316}C$_{NEM}$MVTTL^{322}C$_{4\text{-}VP}$^{323}C$_{4\text{-}VP}$GKNPLGDDEASTTVSK

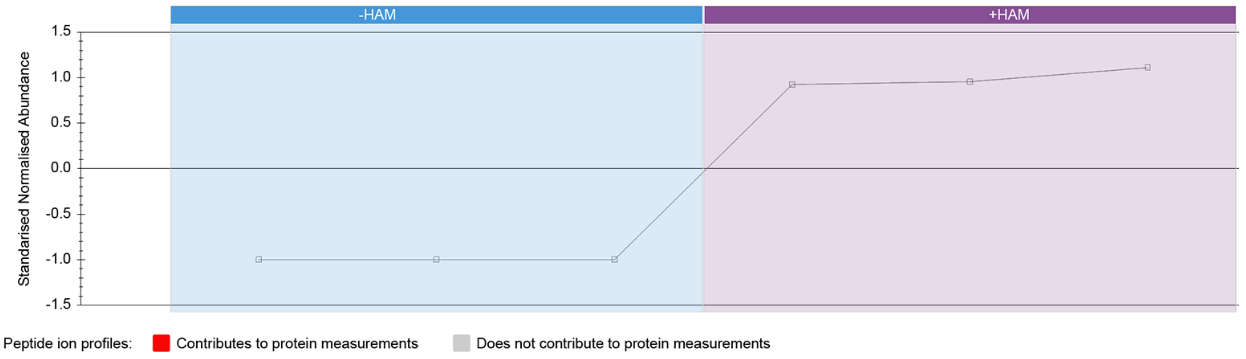

B CRALBP: DHGPVFGP^{38}C$_{NEM}$SQLPR and ^{138}C$_{NEM}$TVEAGYPGVLSTR

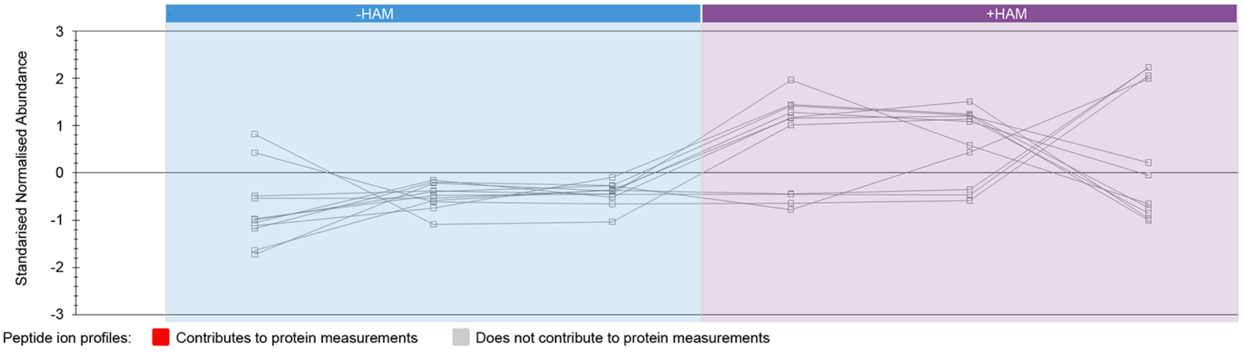

Figure *S6*

Rhodopsin (palmitoylated protein)

Peptides	Synapt G2-Si HDMS^{E}	AB Sciex6600 w/SelexION
K.QFRNCMVTTL$_{322}$C$_{323}$CG<br>KNPLGDDEASTTVSK.T	$_{322}$C: 4VP<br>$_{323}$C: 4VP	$_{322}$C: 4VP<br>$_{323}$C: 4VP

CRALBP (non-palmitoylated protein)

Peptides	Synapt G2-Si HDMS^{E}	AB Sciex6600 w/SelexION
K.DHGPVFGP$_{38}$CSQLPR.H	$_{38}$C: NEM	$_{38}$C: NEM
R.$_{138}$CTVEAGYPGVLSTR.D	$_{138}$C: NEM	$_{138}$C: NEM

RPE65

Peptides	Synapt G2-Si HDMS^{E}	AB Sciex6600 w/SelexION
R.IVITEFGT$_{106}$CAFPDP$_{112}$CK.N	$_{106}$C: NEM<br>$_{112}$C: NEM; 4VP	$_{106}$C: NEM<br>$_{112}$C: 4VP
R.GVEVTDNALVNIYPVGEDY<br>YA$_{146}$CTETNFITK.V	$_{146}$C: 4VP	$_{146}$C: NEM
K.QVDL$_{169}$CNYVSVNGATAHP<br>HIENDGTVYNIGN$_{195}$CFG.K	$_{169}$C: NEM<br>$_{195}$C: NEM	$_{169}$C: NEM<br>$_{195}$C: NEM

Figure *S7*

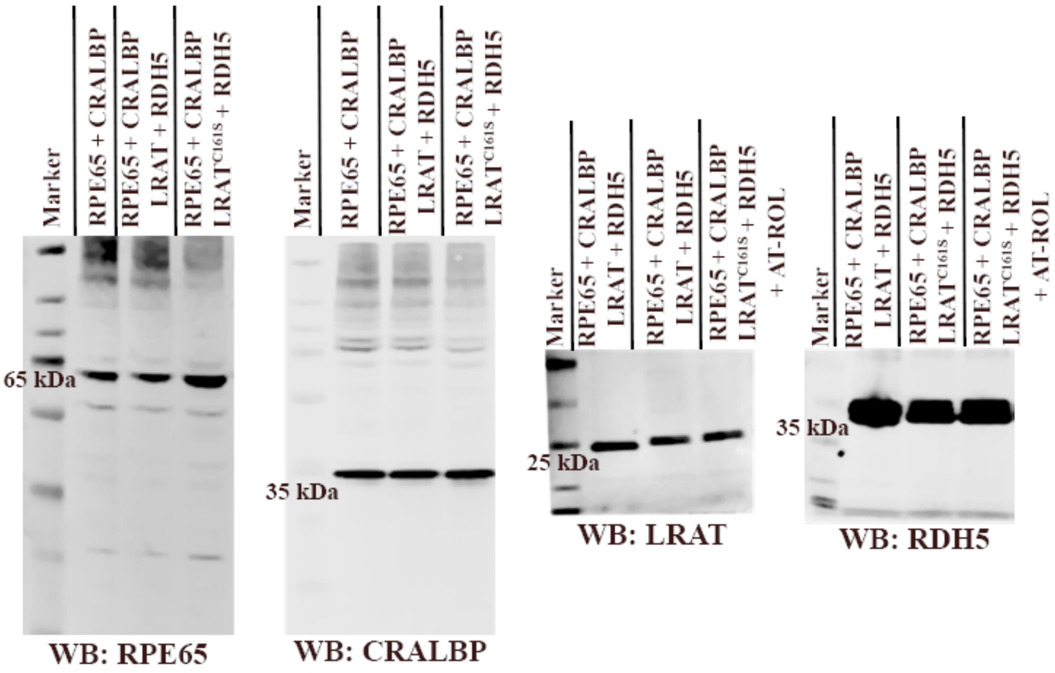

Marker
RPE65 + CRALBP
RPE65 + CRALBP LRAT + RDH5
RPE65 + CRALBP LRAT^C161S + RDH5
65 kDa
WB: RPE65
Marker
RPE65 + CRALBP
RPE65 + CRALBP LRAT + RDH5
RPE65 + CRALBP LRAT^C161S + RDH5
35 kDa
WB: CRALBP
Marker
RPE65 + CRALBP LRAT + RDH5
RPE65 + CRALBP LRAT + RDH5
RPE65 + CRALBP LRAT^C161S + RDH5 + AT-ROL
25 kDa
WB: LRAT
Marker
RPE65 + CRALBP LRAT + RDH5
RPE65 + CRALBP LRAT^C161S + RDH5
RPE65 + CRALBP LRAT^C161S + RDH5 + AT-ROL
35 kDa
WB: RDH5

Figure *S8*

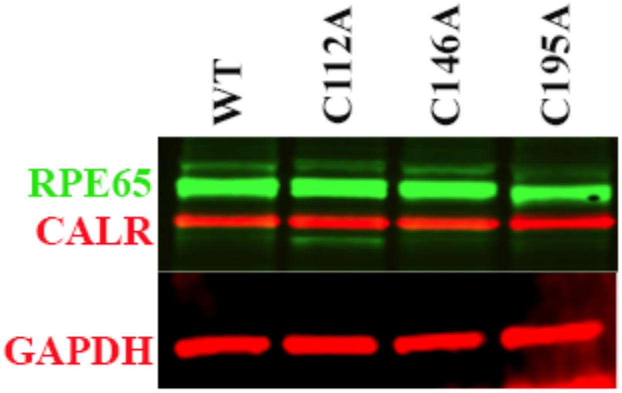

Figure S9

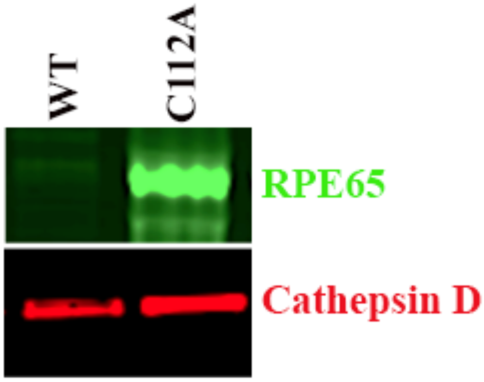

Figure *S10*

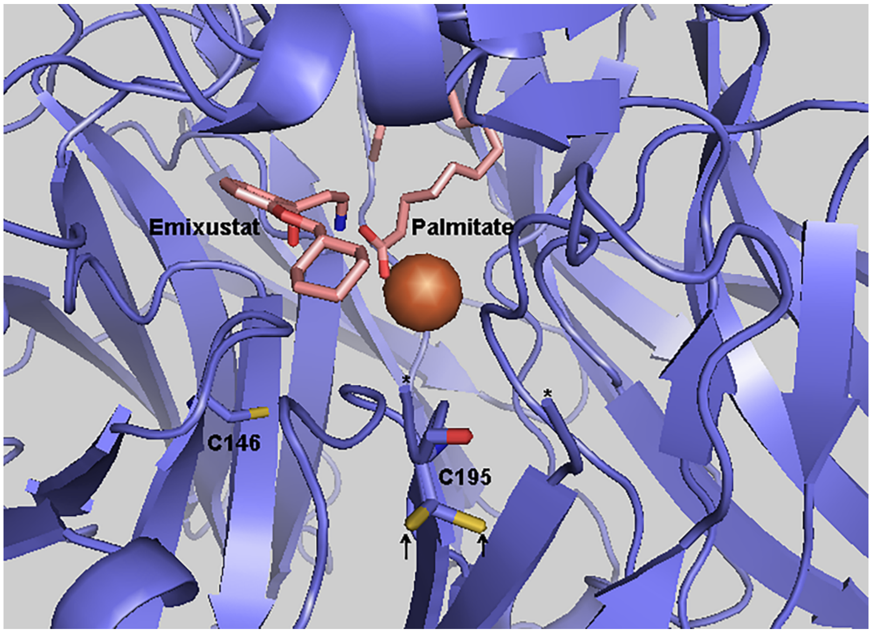

Figure *S11*

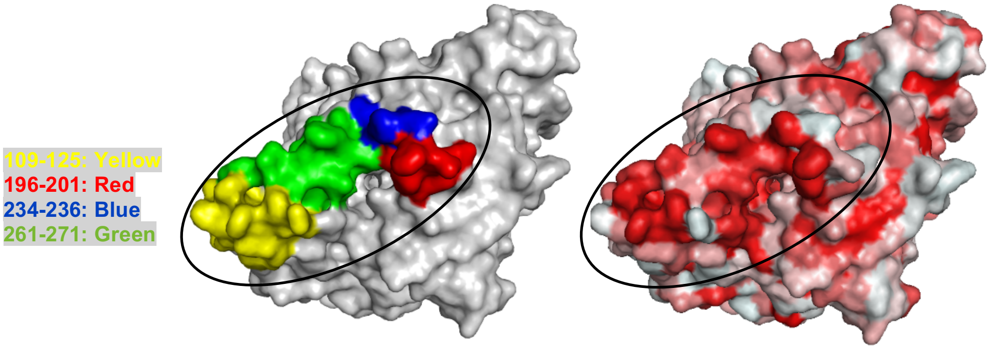

Figure *S12*

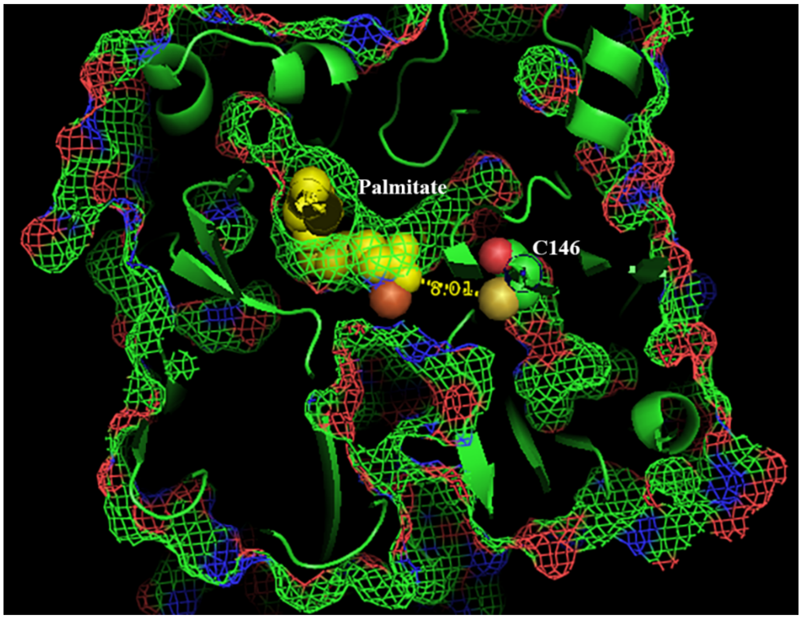

Figure *S13*

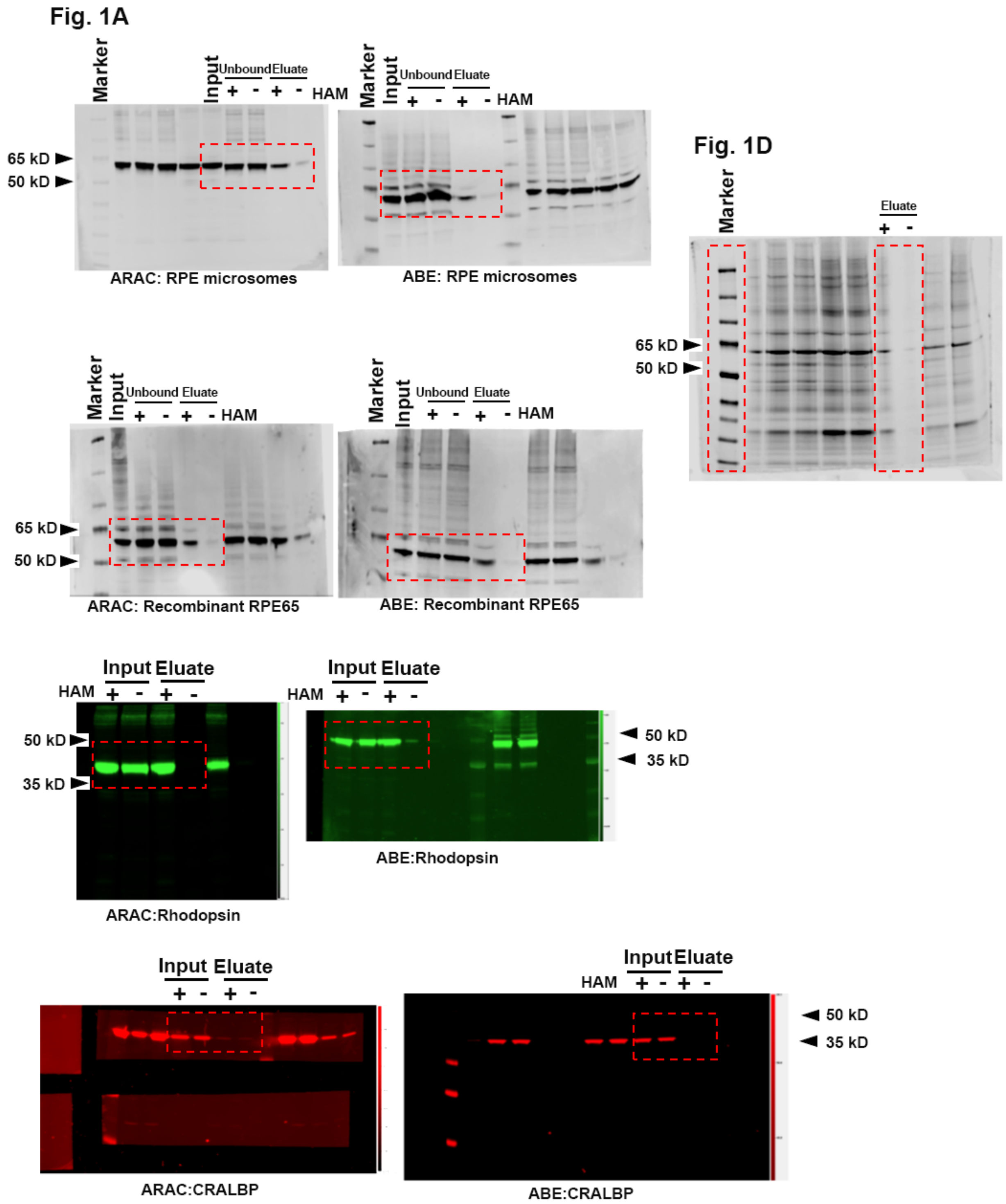

Fig. 1A
Marker
Input
Unbound
Eluate
+ - + -
HAM
65 kD
50 kD
ARAC: RPE microsomes
ABE: RPE microsomes
Fig. 1D
Eluate
ARAC: Recombinant RPE65
ABE: Recombinant RPE65
Input
Eluate
35 kD
ARAC:Rhodopsin
ABE:Rhodopsin
ARAC:CRALBP
ABE:CRALBP

Fig. 2B

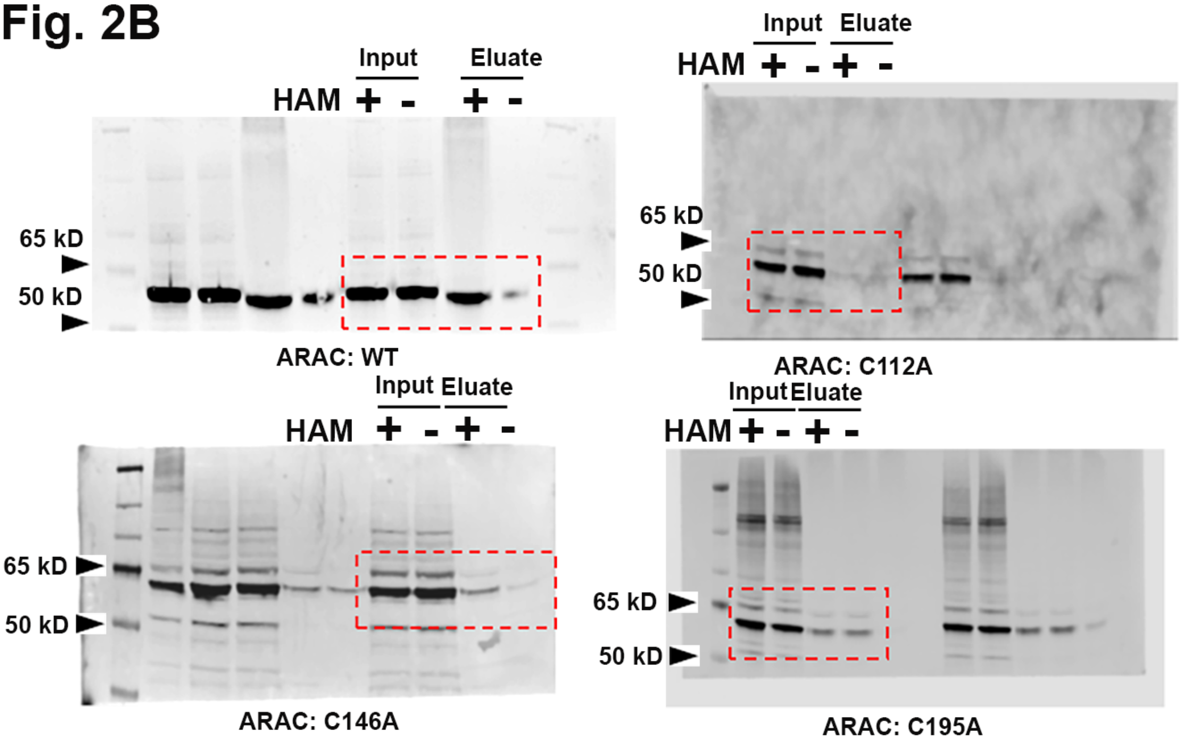
Input
Eluate
HAM + - + -
65 kD
50 kD
ARAC: WT
Input
Eluate
HAM + - + -
65 kD
50 kD
ARAC: C112A
Input
Eluate
HAM + - + -
65 kD
50 kD
ARAC: C146A
Input
Eluate
HAM + - + -
65 kD
50 kD
ARAC: C195A

Fig.5A

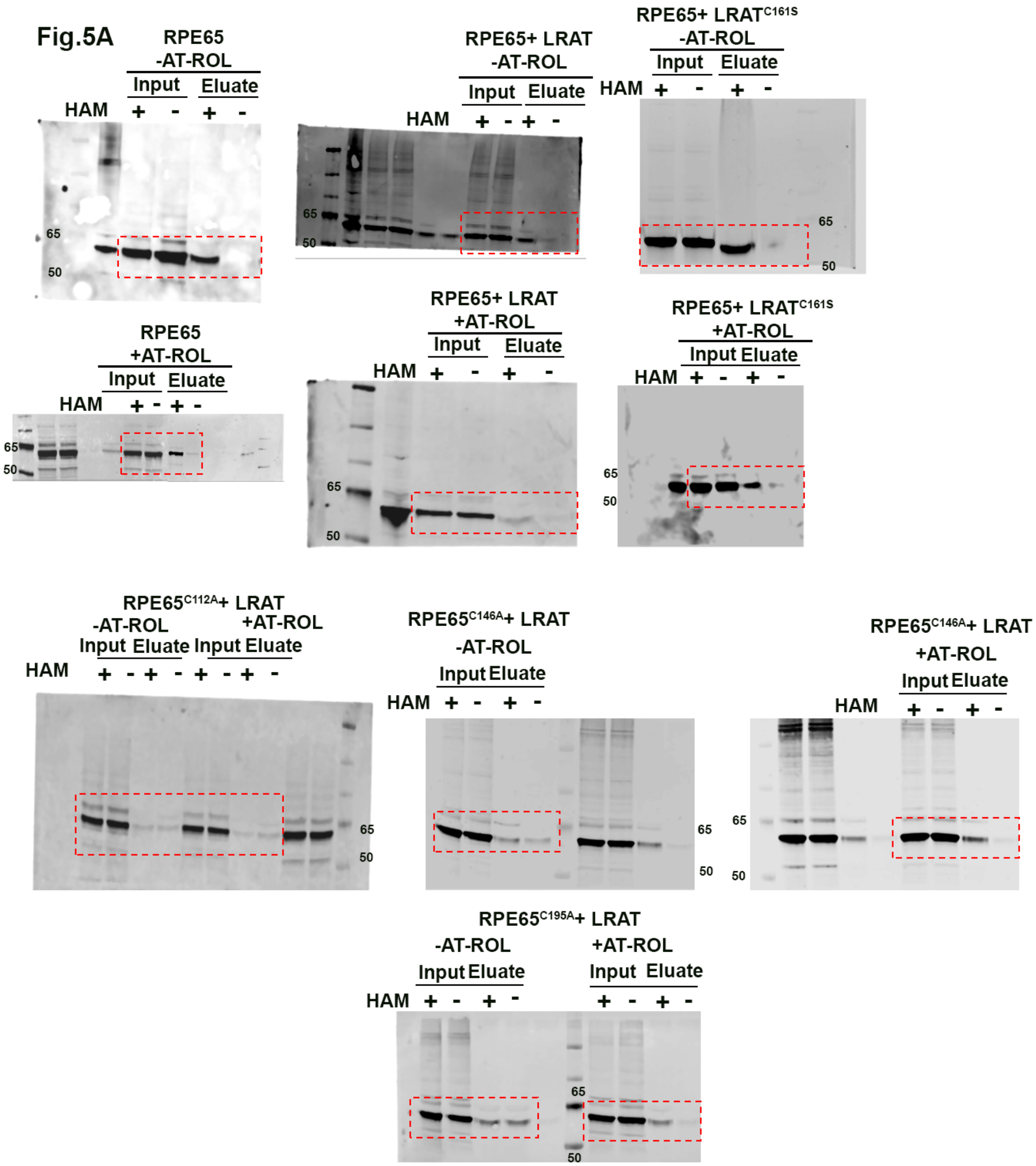
RPE65
-AT-ROL
Input Eluate
HAM + - + -
65
50
RPE65+ LRAT
-AT-ROL
Input Eluate
HAM + - + -
65
50
RPE65+ LRAT^C161S
-AT-ROL
Input Eluate
HAM + - + -
65
50
RPE65
+AT-ROL
Input Eluate
HAM + - + -
65
50
RPE65+ LRAT
+AT-ROL
Input Eluate
HAM + - + -
65
50
RPE65+ LRAT^C161S
+AT-ROL
Input Eluate
HAM + - + -
65
50
RPE65^C112A+ LRAT
-AT-ROL
+AT-ROL
Input Eluate Input Eluate
HAM + - + - + - + -
65
50
RPE65^C146A+ LRAT
-AT-ROL
Input Eluate
HAM + - + -
65
50
RPE65^C146A+ LRAT
+AT-ROL
Input Eluate
HAM + - + -
65
50
RPE65^C195A+ LRAT
-AT-ROL
Input Eluate
+AT-ROL
Input Eluate
HAM + - + - + - + -
65
50

Fig. 6A

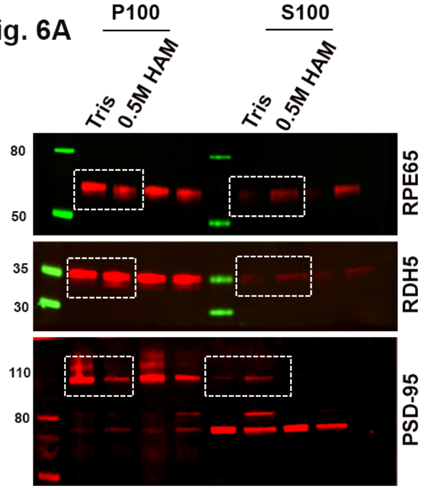

Fig. 6B

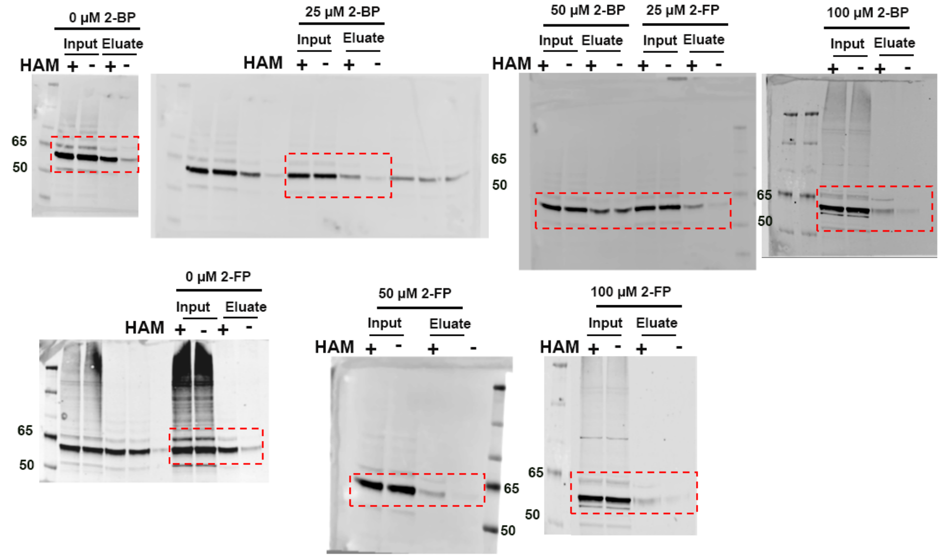

Fig. 6C

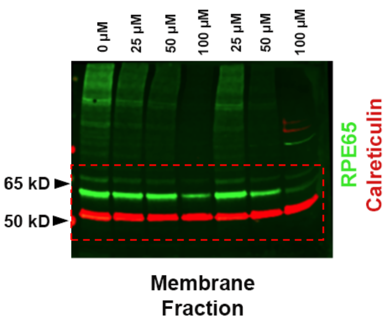

Fig. 6D

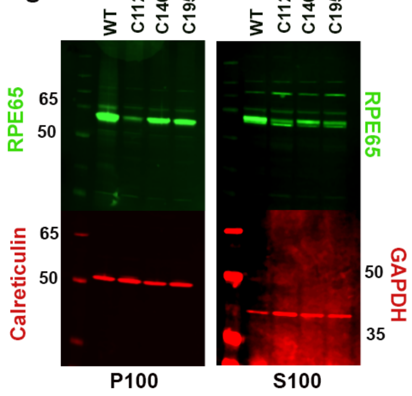

Fig. S1

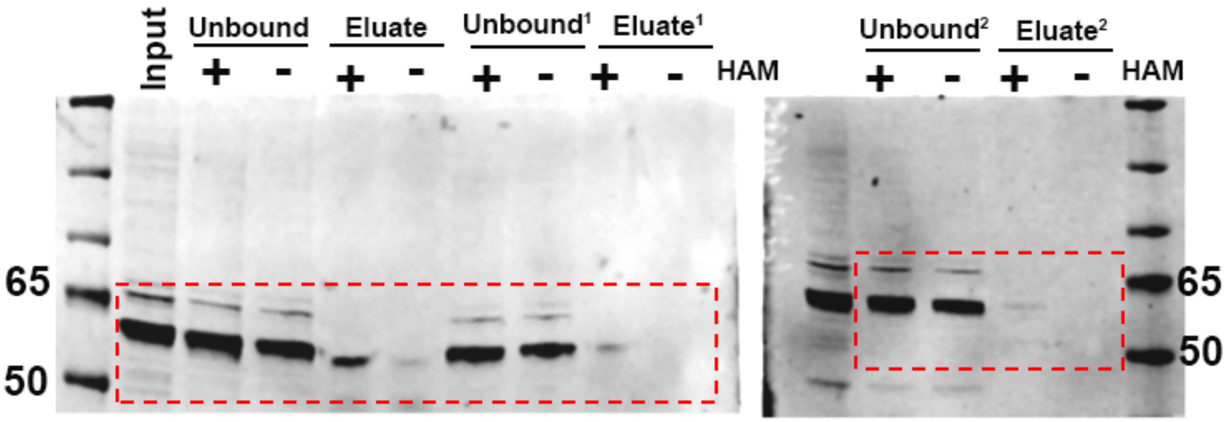

Input
Unbound
Eluate
Unbound1
Eluate1
+
-
HAM
Unbound2
Eluate2
65
50

Fig. S4A

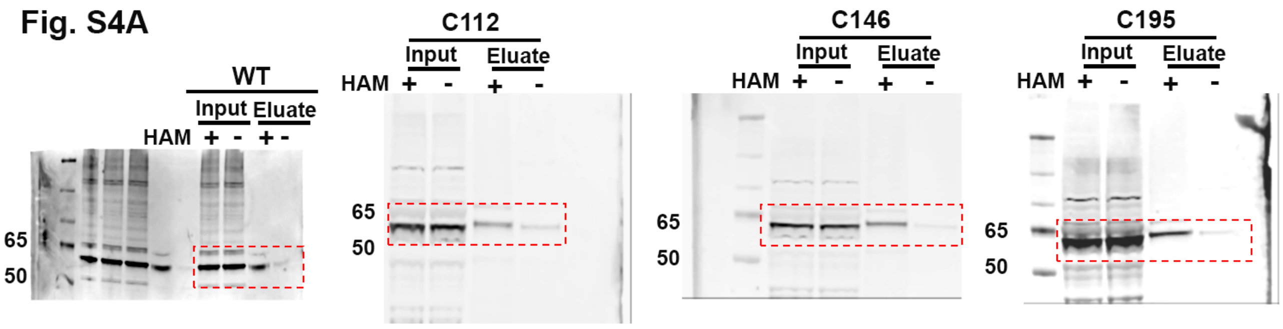

Fig. S4B

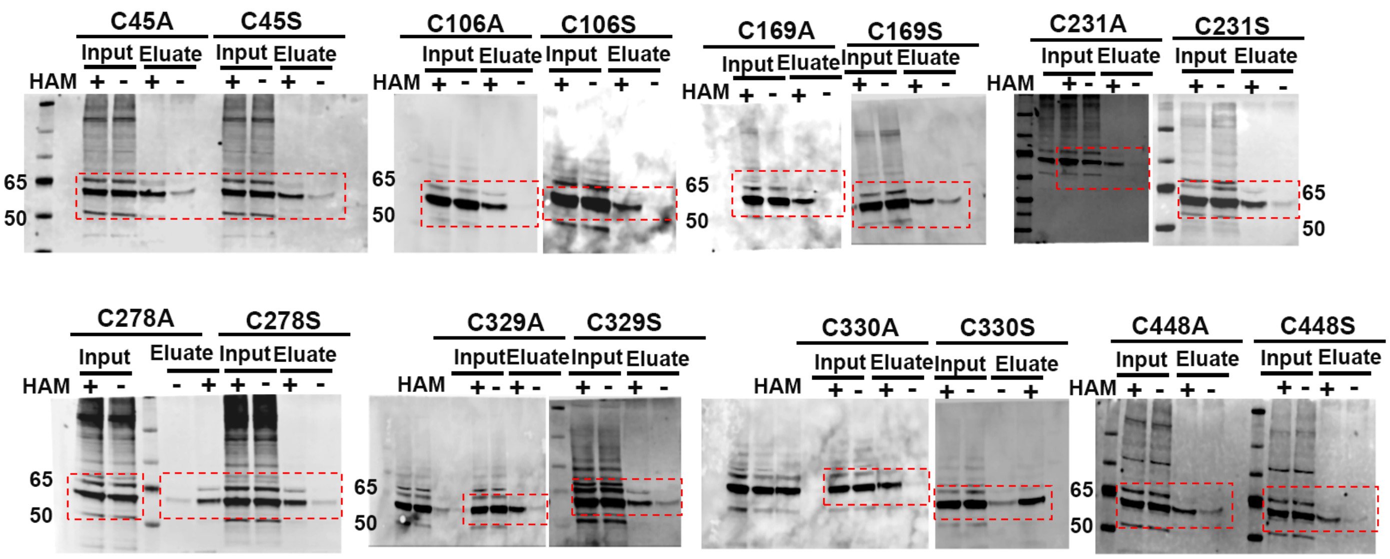

Fig. S4C

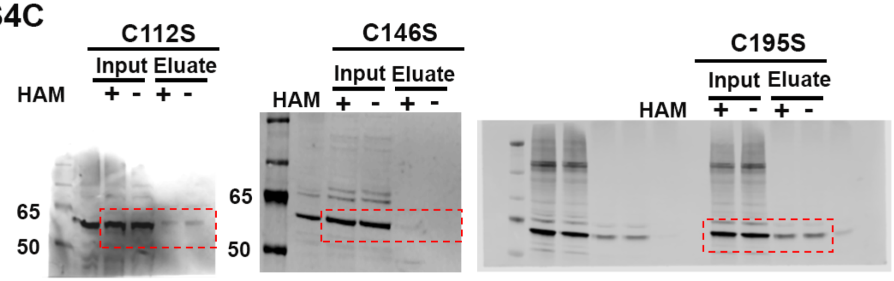

Fig. S8

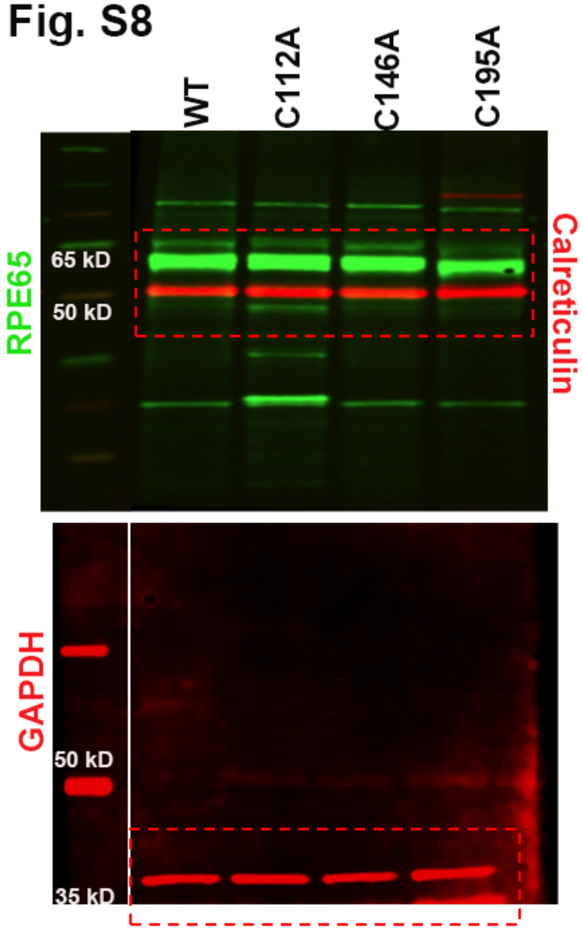
WT
C112A
C146A
C195A
RPE65
65 kD
50 kD
Calreticulin
GAPDH
50 kD
35 kD

Fig.S9

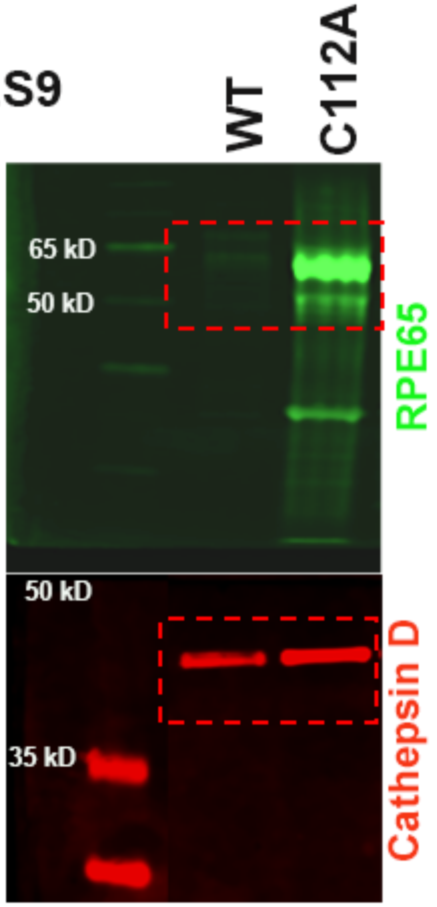
WT
C112A
65 kD
50 kD
RPE65
50 kD
35 kD
Cathepsin D
